# Structural investigation of CDCA3‐Cdh1 protein–protein interactions using *in vitro* studies and molecular dynamics simulation

**DOI:** 10.1002/pro.4572

**Published:** 2023-02-14

**Authors:** Tabassum Khair Barbhuiya, Mark Fisher, Eric D. Boittier, Emma Bolderson, Kenneth J. O'Byrne, Derek J. Richard, Mark Nathaniel Adams, Neha S. Gandhi

**Affiliations:** ^1^ Centre for Genomics and Personalised Health, and School of Chemistry and Physics, Faculty of Science Queensland University of Technology Brisbane Queensland Australia; ^2^ Cancer and Ageing Research Program Woolloongabba Queensland Australia; ^3^ Centre for Genomics and Personalised Health, and School of Biomedical Sciences, Faculty of Health Queensland University of Technology Kelvin Grove Queensland Australia; ^4^ Department of Chemistry University of Basel Basel Switzerland

**Keywords:** anaphase‐promoting complex/cyclosome, CDCA3, Cdh1, co‐immunoprecipitation, molecular dynamics simulation, pairwise energy decomposition

## Abstract

The anaphase‐promoting complex/cyclosome (APC/C) ubiquitin ligase and its cofactor, Cdh1, regulate the expression of several cell‐cycle proteins and their functions during mitosis. Levels of the protein cell division cycle‐associated protein 3 (CDCA3), which is functionally required for mitotic entry, are regulated by APC/C^Cdh1^. CDCA3 is an intrinsically disordered protein and contains both C‐terminal KEN box and D‐box recognition motifs, enabling binding to Cdh1. Our previous findings demonstrate that CDCA3 has a phosphorylation‐dependent non‐canonical ABBA‐like motif within the linker region bridging these two recognition motifs and is required for efficient binding to Cdh1. Here, we sought to identify and further characterize additional residues that participate within this ABBA‐like motif using detailed *in vitro* experiments and *in silico* modeling studies. We identified the role of H‐bonds, hydrophobic and ionic interactions across the CDCA3 ABBA‐like motif in the linker region between KEN and D‐box motifs. This linker region adopts a well‐defined structure when bound to Cdh1 in the presence of phosphorylation. Upon alanine mutation, the structure of this region is lost, leading to higher flexibility, and alteration in affinities due to binding to alternate sites on Cdh1. Our findings identify roles for the anchoring residues in the non‐canonical ABBA‐like motif to promote binding to the APC/C^Cdh1^ and regulation of CDCA3 protein levels.

## 1 INTRODUCTION

The anaphase‐promoting complex/cyclosome (APC/C) is a RING‐type E3 ubiquitin ligase that regulates cellular mitosis by targeting the cell cycle proteins for proteasomal degradation (Yamano 2019). The APC/C associates with two substrate recognition proteins, either Cdc20 or Cdh1, also known as co‐activators, in cell‐cycle dependent manner. Cdc20 binds the APC/C and is required for mitosis onset and during pro‐metaphase to metaphase, whereas Cdh1 is functional from late metaphase through to mitotic exit and is inactivated during G1 (Yamano 2019; Acquaviva and Jonathan 2006). Cdc20 and Cdh1 recognize their substrates via short, disordered peptide sequences, called degrons, namely the D‐box, ABBA motif, and KEN box (He et al. 2013; Di Fiore et al. 2015). These degrons have been identified in cell cycle regulatory proteins and APC/C substrates including APC/C‐Cdh1 modulator 1 (Acm1), BUB1, BUBR1, and Cyclin A (Di Fiore et al. 2015).

**FIGURE 1:**
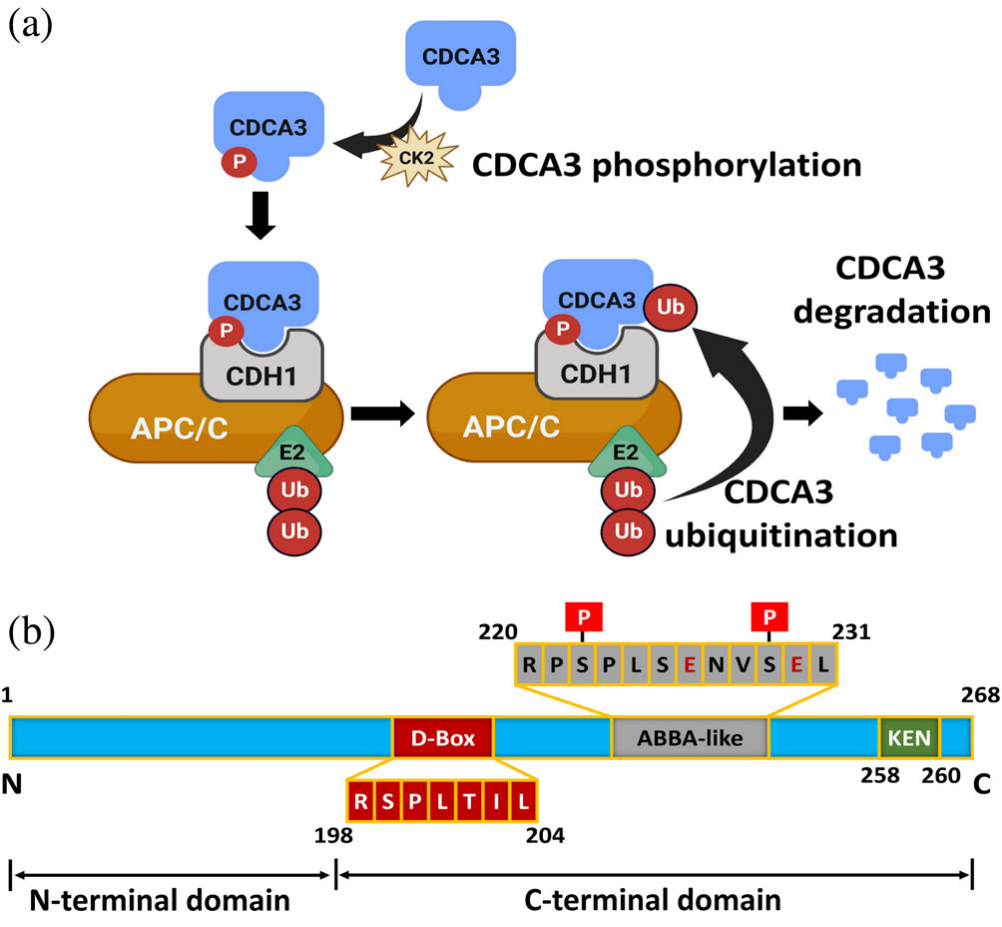
Mechanistic representation of APC/C^Cdh1^ mediated CDCA3 degradation and domain organization of CDCA3. (a) Phosphorylation of CDCA3 by CK2 leads to formation of ABBA‐like motif, that mediates the interaction between CDCA3 and Cdh1 in APC/C complex. Formation of CDCA3‐APC/C^Cdh1^ complex cause ubiquitinylating of CDCA3 and thereby its degradation. Image prepared using BioRender (b) The structure of CDCA3 consist of N‐and C‐terminal domains. The C‐terminal domain, considered in current study consist of well‐characterized D‐box and KEN motifs. It also forms a non‐classical ABBA‐like motif after phosphorylation by CK2, in the linker region between D‐box and KEN motifs.

Cell division cycle associated protein‐3 (CDCA3) is another APC/C substrate. Deregulated expression of CDCA3 is associated with solid malignancies including gastric cancer (Yu et al. 2020; Zhang et al. 2019a), liver cancer (Hu et al. 2015), breast cancer (Pérez‐Peña et al. 2017; Phan et al. 2018), oral squamous cell carcinoma tissues (Uchida et al. 2012), non‐small cell lung cancer (Adams et al. 2017), prostate cancer (Chen et al. 2013), and colorectal cancer (Qian et al. 2018; Zhang et al. 2018). CDCA3 is reported to modulate cell cycle progression from G2 phase to mitosis (Ayad et al. 2003). We have demonstrated in non‐small cell lung cancer (NSCLC) the role of CDCA3 in mediating efficient G2/M progression, tumor cell proliferation, where depleting this protein induces senescence (Kildey et al. 2021). Furthermore, in NSCLC, the levels of CDCA3 are regulated by APC/C^Cdh1^‐mediated degradation. The degradation of CDCA3 was also found to be phosphorylation‐dependent and is mediated by casein kinase (CK2) (Kildey et al. 2021; Sahin et al. 2021). Figure 1a represents the schematic of the phosphorylation‐mediated association of CDCA3 with APC/C^Cdh1^ and its degradation.

The structure of CDCA3 consists of N‐ and C‐terminal regions. While CDCA3 is an intrinsically disordered protein (IDP), this protein has two well‐characterized C‐terminus degrons, a non‐structured D‐box (R‐X‐X‐L) and a KEN box, starting at Arg198 and Lys258, respectively. Cdh1 recognizes the residues within the KEN and D‐box motifs of CDCA3 via respective H‐bond and hydrophobic interactions. Although CDCA3 lacks a classical ABBA motif, we have previously identified that phosphorylation‐mediated generation of negative charges at Ser222 and Ser229 form a non‐canonical ABBA‐like motif (Arg220‐Leu231) (Kildey et al. 2021). We also identified the presence of hydrophobic residues at a similar position to other APC/C substrates such as BUBR1, which at least for CDCA3, plays a crucial role in promoting interaction with Cdh1 to enable its degradation. Apart from the two phospho‐sites, the negatively charged Glu226 and Glu230 are also conserved in other substrates, responsible for interaction with the positive residues of Cdh1 (Kildey et al. 2021). Figure 1b represents the structural domains of CDCA3 containing three degrons: D‐box, ABBA‐like, and KEN box motifs. We previously identified a non‐classical ABBA‐like motif in the C‐terminal domain of CDCA3. However, the role of amino acid residues within the motif, mediating interaction with Cdh1 and the structural implications of ABBA‐like motif formation were poorly understood.

In our present study, we sought to further define the non‐canonical ABBA‐like motif of CDCA3 using co‐immunoprecipitation (Co‐IP) and classical molecular dynamics (MD) simulations to understand structural dependencies upon each residue for interaction with Cdh1. Our data verify the importance of two phosphorylation sites at Ser222 and Ser229 sites to generate the non‐canonical ABBA‐like motif. Furthermore, other hydrophobic residues within this motif are required for efficient Cdh1 binding. Our unbiased MD simulation also point to possible alternate binding modes for CDCA3 mutants highlighting the reliance on key residues within the ABBA‐like motif for efficient Cdh1 binding.

## 2 RESULTS

### 2.1 *In vitro* study identifies ionic and hydrophobic residues of CDCA3 impacting Cdh1 binding

**FIGURE 2:**
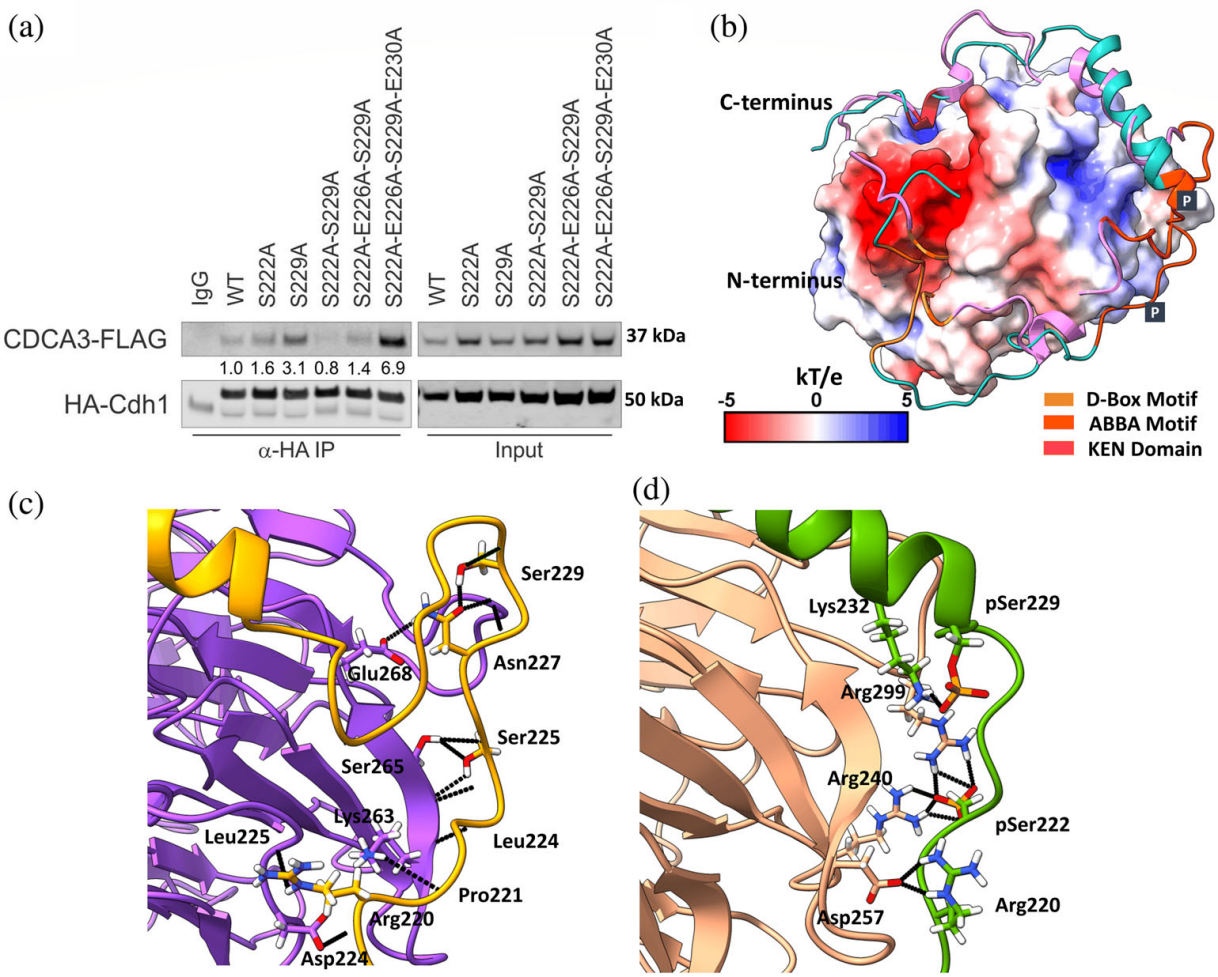
Interaction of CDCA3 C‐terminal domain with Cdh1. (a) Immunoprecipitation assay of HA‐Cdh1 with wildtype (WT) or alanine mutants of Flag‐tagged CDCA3 expressed in H460 cells. Numbers indicate the efficiency of CDCA3‐FLAG mutants' precipitation by HA‐Cdh1 relative to the WT‐CDCA3 signal. The IP is representative of three independent experiments (*n* = 3), (b) molecular electrostatic potential surface (−5 to +5 kT/e) representation of Cdh1 and WT‐CDCA3 (shown in the square‐edged pink ribbon) overlaid with the same sequence (shown in the round‐edged cyan ribbon), phosphorylated at Ser222 and Ser229 at the ABBA‐like motif (pS222 + pS229). Both the sequences consist of D‐box (yellow), ABBA‐like motif (orange) and KEN domain (crimson). The ABBA‐like motif of the phosphorylated sequence occupies a slightly different binding site than the WT sequence, at its N‐terminus, (c) the molecular interaction diagram of WT‐CDCA3 peptide shows the important ABBA‐motif residues interacting with the Cdh1 binding site via H‐bond interactions, shown in black. (d) The molecular interaction of the phosphorylated (pS222 + pS229) ABBA‐like motif with the Cdh1 shows the involvement of charged residues, Arg220 and pSer229 with Aspartic acid and Arginine, respectively, via H‐bond and salt‐bridge formation.

To investigate the contribution of residues within the CDCA3 non‐classical ABBA‐like motif that might impact Cdh1 binding, we mutated CDCA3 and performed immunoprecipitation analysis with Cdh1. We evaluated single (S222A and S229A) and dual mutation of the known CDCA3 phosphorylation sites, S222A and S229A (2X) and the role of phospho‐mimic, two glutamic acid residues, S222A‐E226A‐S229A (3X) and S222A‐E226A‐S229A‐E230A (4X) by mutating them to non‐phosphorylatable alanine mutants. These residues were selected given that they are conserved in the ABBA motif of other Cdh1 substrates. As shown in Figure 2a, the dual mutation of Ser222 and Ser229, each as phosphorylation sites, markedly impaired the association between Flag‐CDCA3 and HA‐Cdh1, consistent with our previous study, suggesting that both phospho‐sites are required for efficient binding to Cdh1 surface via ionic interactions (Kildey et al. 2021). Of the individual mutations relative to wildtype (WT)‐ CDCA3, S229A exhibited an enhanced association with Cdh1 versus S222A, pointing to a significant contribution for phosphorylated Ser222 (pS222) in the interaction with Cdh1 over Ser229. The triple mutant (3X) demonstrated a similar association pattern as WT‐CDCA3. However, the additional incorporation of another mutation, E230A, into the 3X mutant (termed 4X) enhanced the association between the two proteins. Together, this reflects the predominance of hydrophobic interactions induced by masking all the charged residues in the ABBA‐like motif of CDCA3 with alanine.

### 2.2 *In silico* evaluation of CDCA3 ABBA‐like motif binding to Cdh1

Given our immunoprecipitation analysis demonstrated the involvement of hydrophobic interactions within the CDCA3 ABBA‐like motif, we next sought to examine the interaction patterns with Cdh1 using MD simulation studies. The CDCA3 C‐terminus (Ser193‐Glu267) containing D‐box, ABBA‐like motif, and KEN box domain was evaluated for binding to the respective binding sites within Cdh1 using MD simulations and free energy calculations. Considering the importance of phosphorylated residues within the ABBA‐like motif, we accounted for these post‐translational modifications (PTMs) in our modeling of the CDCA3 peptide. As such, we examined pS222 + pS229 (di‐phosphorylated CDCA3), S222A + pS229 (Ser222 point mutant with phosphorylated Ser229), and pS222 + S229A (Ser229 point mutant with phosphorylated Ser222) relative to wildtype CDCA3 (represented as WT, which is unphosphorylated). We have also considered two more systems where the point mutants have one phospho‐site free (S222A and S229A). The molecular‐mechanics General Born surface area (MM‐GBSA) methods have previously been used to calculate the free energy of binding to study the protein–protein interactions (Chen et al. 2016; Weng et al. 2019). We have used a similar approach to assess and compare the binding free energies of Cdh1 interacting with different CDCA3 C‐terminal domains. The MM‐GBSA binding free energies (Δ*G*_Total_) of the last 300 ns simulations of each CDCA3‐Cdh1 complex pair, summarized in Table 1, demonstrates a stronger interaction of the di‐phosphorylated system with the Cdh1 WD40 domain compared with the unphosphorylated form of WT CDCA3. Further insights into the individual energy components suggest that the major driving force for the increased binding affinity of the di‐phosphorylated system can be attributed to the increased electrostatic (*E*_Elec_) and hydrophobic (*E*_*V*d*W*_) interactions with the energy difference of ~ −110 kcal/mol and ~ −16 kcal/mol, respectively between WT and di‐phosphorylated CDCA3 simulation system. However, the increased negative binding free energy arising due to the *E*_Elec_ is compensated by the polar solvation free energy (*E*_GB_). The sum of these two terms is ~6 kcal/mol more positive in the di‐phosphorylated CDCA3 than the WT sequence. This suggests overall higher contribution of the electrostatic and hydrophobic interactions towards its total binding free energy of the di‐phosphorylated CDCA3.

**TABLE 1:** The binding free energy components and standard deviations calculated with MM‐GBSA‐based endpoint method using the structures generated with 300 ns MD simulation.

Simulation system	Binding free energies (kcal/mol)							
	*E*_*V*d*W*_	*E*_Elec_	*E*_GB_	*E*_Surf_	Δ*G*_Gas_	Δ*G*_Solv_	Δ*G*_Total_	SD(±)
WT	−226.67	−777.23	854.22	−32.62	−1003.91	821.60	−182.30	8.29
pS222 + pS229	−242.49	−886.78	970.21	−36.35	−1129.27	933.86	−195.41	5.05
S222A + pS229	−225.06	−879.46	943.59	−33.00	−1104.51	910.59	−193.92	6.37
pS222 + S229A	−230.74	−866.58	937.04	−34.34	−1097.32	902.70	−194.62	20.11
S222A	−239.06	−791.72	876.53	−34.71	−1030.78	841.82	−188.96	12.58
S229A	−243.83	−818.65	895.30	−35.68	−1062.49	859.62	−202.87	14.83
2X	−247.00	−838.98	903.55	−36.71	−1085.98	866.84	−219.14	6.21
3X	−231.24	−880.14	948.39	−33.72	−1111.38	914.67	−196.71	4.26
4X	−230.94	−762.64	833.33	−33.36	−993.58	799.97	−193.61	9.85

*Note*: The terms, *E*_*V*d*W*_, *E*_Elec_, *E*_GB_, and *E*_Surf_ refer to the energy contribution due to van der Waals interactions, electrostatic interactions, polar and non‐polar components of solvation free energies, respectively. Δ*G*_Gas_ represents the sum of *E*_*V*d*W*_ and *E*_Elec_ as the ΔEMM component in the gaseous state, whereas Δ*G*_Solv_ represents the summation of *E*_GB_ and *E*_Surf_ components. Δ*G*_Total_ refers to the binding free energy for the Cdh1 with the C‐terminal domain of WT‐CDCA3 and its mutants. SD represents the standard deviations of the Δ*G*_Total_ of three simulation repeats of each system.

We also performed the clustering analysis of MD simulation trajectories of the last 30,000 frames of each simulation system, corresponding to 300 ns and investigated the interactions of ABBA‐like motifs with their respective binding sites at the Cdh1 interface. The top cluster and the representative frame of each simulation are displayed in Table 2. As shown in Figure 2b, representative frames from the top cluster of the simulation trajectories are displayed as overlaid models of both WT and di‐phosphorylated C‐terminal of CDCA3 (residues Ser193‐Glu267), interacting with Cdh1. The investigation of secondary structure across the three specific domains of the WT‐CDCA3 demonstrates the prevalence of an unstructured form with some structural bends and turns across few residues throughout the domain (Figure S1a), one of the characteristics of interaction of IDP with the partner protein. However, phosphorylation within the ABBA‐like motif of di‐phosphorylated CDCA3 induces the formation of prominent stable helical structures at the C‐terminus of the ABBA‐like motif beginning at the residue, pSer229 (linker region between KEN and D‐box recognition motifs) that remains throughout the simulation timeframes (Figure S1b).

**TABLE 2:** Clustering analysis of the simulation systems using DB SCAN clustering algorithm‐ to segregate the most populated cluster from the simulation trajectories.

Simulation system	No. of frames in top cluster (C#0)	Percentage of frames in top cluster (%)	Average distance ± Stdev (Å)	Centroid frame (#)	AvgCDist (Å)	DBI	pSF
WT	5228	17.4	1.61 ± 0.32	12,585	1.47	1.60	2749.38
pS222 + pS229	2994	10.0	1.65 ± 0.17	19,724	1.45	1.95	1016.54
S222A + pS229	2671	8.9	1.46 ± 0.26	10,120	1.54	1.92	1258.70
pS222 + S229A	6120	20.4	1.29 ± 0.21	16,840	1.56	1.72	2672.43
S222A	4172	13.9	1.28 ± 0.19	2,651	2.89	1.93	3174.58
S229A	7849	26.2	1.22 ± 0.23	28,666	1.90	1.96	2037.70
2X	4802	16.0	1.63 ± 0.33	17,586	1.61	1.59	3027.09
3X	3460	11.5	1.60 ± 0.29	24,685	1.54	1.81	2517.30
4X	4417	14.7	1.26 ± 0.19	24,391	2.02	1.71	1368.50

*Note*: The clustering analysis of the last 30,000 frames of MD simulation, corresponding to 300 ns, was performed using the BDScan algorithm using minipoints ~5.0 and an epsilon value of 1.0 Å. Column 4 in the table represents the average distance between the points in the top cluster, C#0. The centroid frame of each system represents the structure with the lowest cumulative distance to other frames in the cluster. AvgCDist represents the average distance of the top cluster to every other cluster. The DBI and the pSF column represent the clustering quality. High pSF and low DBI indicate better clustering.

Consistent with the immunoprecipitation analysis, pS222 + S229A exhibited a stronger binding energy with Cdh1 versus S222A + pS229 while the unphosphorylated mutants (S222A and S229A) had higher relative binding free energies than unmodified CDCA3. Amongst the two single phospho‐mutants, the relative higher binding energy of pS222 + S229A is attributed to the increased contribution of the hydrophobic interactions. In contrast to our immunoprecipitation analysis, simultaneous mutation of Ser222 and Ser229 (2X) exhibited enhanced binding relative to the di‐phosphorylated CDCA3, while the 4X‐CDCA3 mutant did not show any impact on binding affinity to Cdh1. However, the triple mutant (3X), which includes the Ser222 and Ser229 mutants, demonstrated comparable binding energy to di‐phosphorylated sequence, aligning with the immunoprecipitation data. The molecular simulations suggested that the triple mutant forms unique hydrophobic interactions with Cdh1, independent of the domain bound by the ABBA‐like motif in wildtype CDCA3 (Figure S2). Interestingly, the 4X mutant peptide system did not show any significant change in binding affinity when compared to the di‐phosphorylated peptide complex with Cdh1, again contradicting the experimental findings.

**FIGURE 3:**
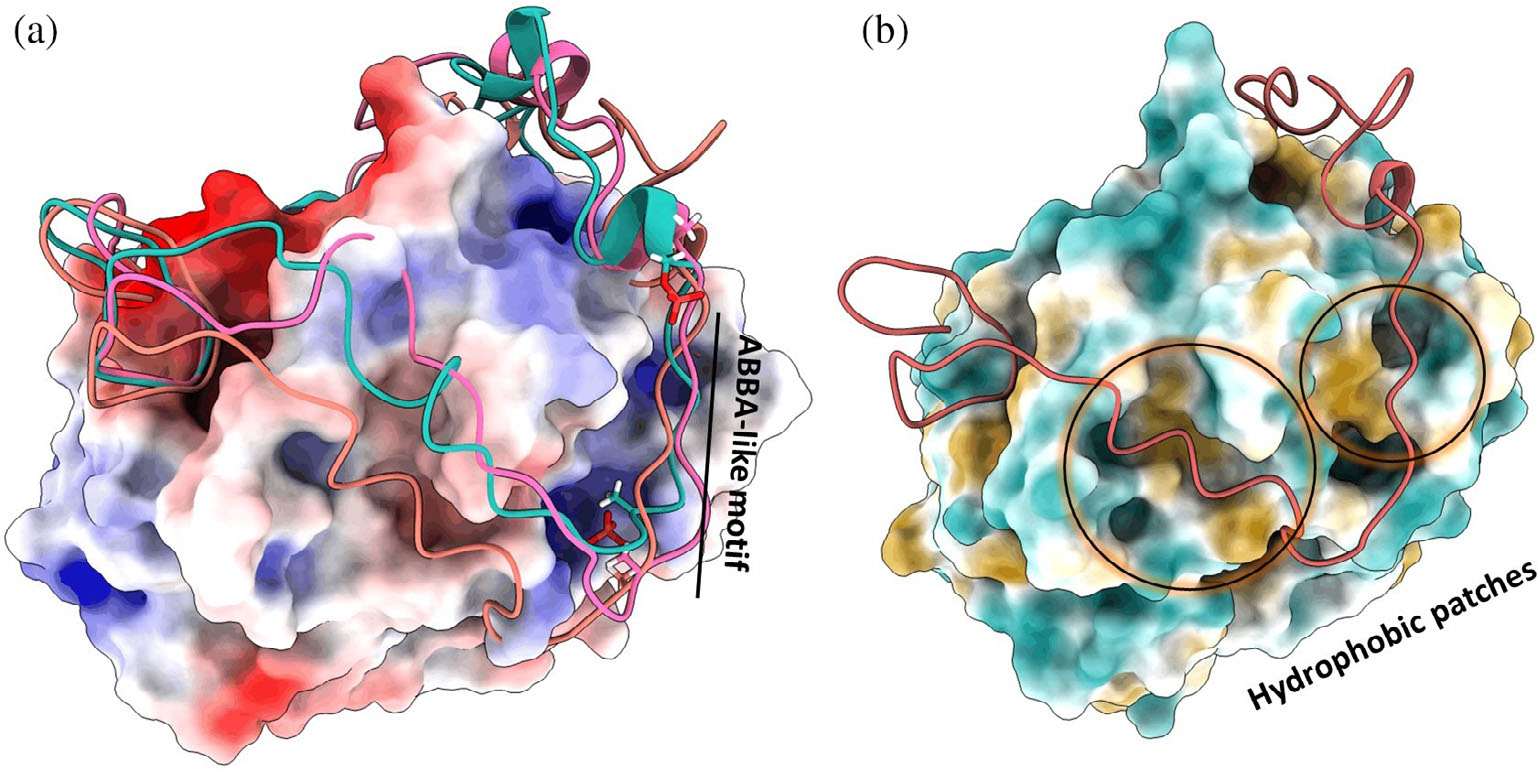
Binding of C‐terminal CDCA3 mutant peptides with Cdh1. (a) The molecular electrostatic potential surface (in kT/e) of the WD40 domain of Cdh1 scaled from electronegative (dark red, −5 kT/e) to white to electropositive interaction (dark blue, 5 kT/e). The superimposed interaction diagram of CDCA3 C‐terminal mutant peptides; pS222 + S229A (light pink) and S222A + pS229 (cyan) and 2X (coral) containing D‐box, ABBA‐like motif, and KEN with Cdh1 WD40 domain. The three mutant peptides show similar binding across the ABBA‐like motif whereas the 2X mutant shows a change in binding residues between D‐box and ABBA‐like motif. (b) The binding of 2X mutant peptide on the molecular lipophilic potential surface (colored from cyan: most hydrophilic to white: neutral to dark goldenrod: most lipophilic, range = −20 to 20) of the Cdh1 WD40 domain shows hydrophobic interactions between resides of ABBA‐like motif and the aliphatic amino acids residues preceding the ABBA‐like motif.

To further assess the contribution of each residue toward the total binding free energy, we investigated the pair‐wise energy decomposition of the interacting domains of WT and the mutated systems with Cdh1. As shown in Figure 2c,d and Figure S3, the ionic interaction within the ABBA‐like motif of di‐phosphorylated CDCA3 predominates due to the interactions between the phosphate group on the side chain of Ser222_CDCA3_ (pS222) and Arg240_Cdh1_ and Arg299_Cdh1_ with binding free energies (Δ*G*) of −16.0 and −12.4 kcal/mol, respectively. Phosphorylation also induces a structural change that leads to another ionic interaction between Arg220_CDCA3_ with Asp257_Cdh1_ with an energy of −5.5 kcal/mol, which was otherwise absent in the WT CDCA3. Mutation of one of the phospho‐site, pS229 to alanine in the pS222 + S229A system leads to increased ionic interactions between the pS222_CDCA3_ with Arg240_Cdh1_ and Arg299_Cdh1_ (Δ*G* increased to −20.0 kcal/mol). This also increased the ionic interactions between the side chains of Arg220_CDCA3_ and Asp257_Cdh1_ with Δ*G* of −17.0 kcal/mol (Figure S3). However, mutation of pS222 to alanine in the S222A + pS229 system, abolishes the ionic interaction at position 222 of CDCA3 and reduces the ionic interaction between Agr220_CDCA3_ and Asp257_Cdh1_ (Δ*G* = −7.5 kcal/mol) compared to the pS222 + S229A system. The presence of a single phospho‐site at Ser229 introduces an ionic interaction between pS229_CDCA3_ and Lys262_Cdh1_ with ΔG of −13.7 kcal/mol, which was otherwise absent or very weak in pS222 + S229A or the di‐phosphorylated system, respectively. This validates the findings that of the two phospho‐sites, pS222 plays a crucial role while interacting with Cdh1 via an ionic interaction, whereas pS229 is predominantly involved in promoting structural changes in the C‐terminal end of the ABBA‐like motif. Although the pS222 + S229A, S222A + pS229, and 2X systems share a similar Cdh1 binding surface (Figure 3a), only pS222 mediates an ionic interaction. Detailed investigation of the 2X mutant shows the formation of hydrophobic interactions with Cdh1 at the residues between the D‐box and ABBA‐like motif in the modeled structure (Figure 3b). The pair‐wise energy decomposition of MM‐GBSA calculation of 2X mutant (Figure S3) indicates interactions of Arg220_CDCA3_ with Glu239_Cdh1_ and Arg240_Cdh1_, respectively. The Pro221_CDCA3_‐Pro223_CDCA3_ also interact with Arg240_Cdh1_ and Glu226_CDCA3_ interacts with Lys262_Cdh1_. The Leu224_CDCA3_ forms hydrophobic interactions with Leu243_Cdh1_ (*E*_*V*d*W*_ = −0.53 kcal/mol), Leu264_Cdh1_ (*E*_*V*d*W*_ = −0.76 kcal/mol) and Ile298_Cdh1_ (*E*_*V*d*W*_ = −0.4 kcal/mol). In case of 4X mutants, Arg220_CDCA3_ form ionic interactions with Asp257_Cdh1_ and Glu282_Cdh1_. The residues from Pro221‐Asn227_CDCA3_ are involved in hydrophobic interactions with Leu264_Cdh1_ (*E*_*V*d*W*_ of Pro223_CDCA3_‐Leu264_Cdh1_ is −0.66 kcal/mol, and Ala226 _CDCA3_‐Leu264_Cdh1_ is −0.65 kcal/mol), Ser265_Cdh1_ (*E*_*V*d*W*_ of Ala226_CDCA3_‐Ser265_Cdh1_ is −0.38 kcal/mol) and Ile298‐Leu303_Cdh1_ (E_VdW_ of Pro223_CDCA3_‐Ile298_Cdh1_ is −0.67 kcal/mol and Ser225_CDCA3_‐Leu303_Cdh1_ is −0.73 kcal/mol).

We also examined the pair‐wise energy decomposition of other two recognition motifs: KEN and D‐box, to understand the change in binding energetics from those residues due to mutagenesis in ABBA‐like motif. The Glu259_CDCA3_ within the KEN domain of WT‐CDCA3 shows strong interactions with Gln401_Cdh1_ (Δ*G* = −9.7 kcal/mol), Thr377_Cdh1_ (Δ*G* = −7.5 kcal/mol) with significant contribution from electrostatic interactions via H‐bond formation. Glu259_CDCA3_ also forms relatively weaker ionic interactions with Arg445_Cdh1_ (Δ*G* = −3.7 kcal/mol) and H‐bond interaction with Gly376_Cdh1_ (Δ*G* = −4.9 kcal/mol) (Figure S4). The Lys258_CDCA3_ interacts with the Asn333_Cdh1_ and Asn335_Cdh1_ with ΔG values of −5.4 and −4.6 kcal/mol, respectively. The Asn260_CDCA3_ imparts strong interactions with Asp187_Cdh1_ and Arg445_Cdh1_ with binding energies, ΔG of −6.4 and −7.9 kcal/mol, respectively and a weaker interaction with Phe188_Cdh1_ (Δ*G* = −2.6 kcal/mol). Similar energy profiles were observed within the KEN domain of di‐phosphorylated and other CDCA3 mutants. In the D‐box motif of WT‐CDCA3 (Figure S5), Arg198_CDCA3_ forms side chain ionic interactions with Asp180_Cdh1_ (Δ*G* = −9.3 kcal/mol), Asp464_Cdh1_ (Δ*G* = −4.0 kcal/mol) and Glu465_Cdh1_ (Δ*G* = −3.5 kcal/mol) and backbone H‐bond interaction Pro182_Cdh1_ (Δ*G* = −1.9 kcal/mol) and Thr466_Cdh1_ (Δ*G* = −3.5 kcal/mol). Di‐phosphorylation increases ionic interactions of Arg198_CDCA3_ with Asp180_Cdh1_ and Glu465_Cdh1_ leading to increase in Δ*G* values to −11.48 and −7.6 kcal/mol, respectively. This also abolishes the interactions of Arg198_CDCA3_ with Asp464_Cdh1_ and Thr466_Cdh1_. Interestingly, in other alanine mutants of CDCA3 ABBA‐like motif (2X, 3X, and 4X), the ionic interaction of Arg198_CDCA3_ with Asp180_Cdh1_ has reduced (ΔG values for 2X, 3X and 4X are −2.0, −3.4, and −4.8 kcal/mol, respectively). Other D‐box residues (Ser199‐Ile203) interact with the hydrophobic amino acids of Cdh1's binding site via H‐bond formation and hydrophobic interactions.

**FIGURE 4:**
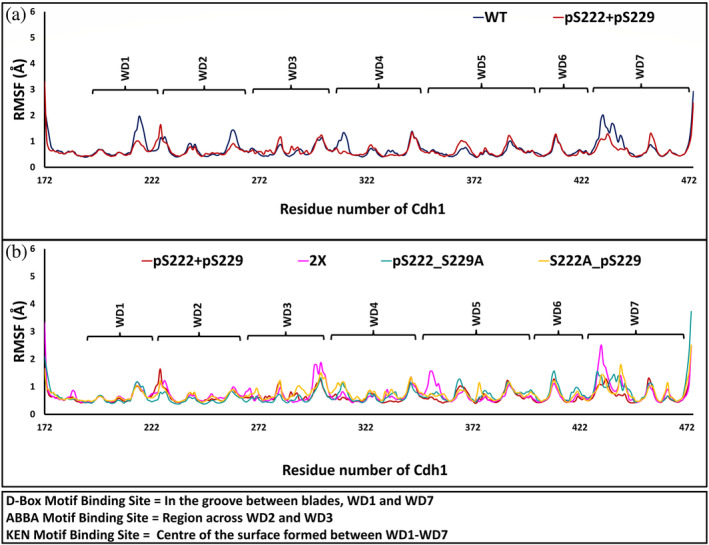
The residue‐by‐residue root mean square fluctuations (RMSF) of the backbone atoms of Cdh1 in different simulation systems. (a) The phospho‐dependent interaction of pS222 + pS229 CDCA3 mutant leads to the stabilization of the Cdh1 backbone across the D‐box motif (WD1 and WD7) and ABBA‐like motif (WD2, WD3, and WD4) binding sites, (b) 2X‐CDCA3 increases the conformational flexibility of the Cdh1 backbone across the D‐box and the ABBA‐like and D‐box liner region.

To evaluate if any conformational changes have been introduced in Cdh1 after binding to CDCA3 mutants, we have determined the root mean square fluctuations (RMSFs) of the backbone atoms of Cdh1, averaged over the last 300 ns simulation trajectories across the domain (Figure 4a,b). The di‐phosphorylation of the WT‐CDCA3 stabilizes the Cdh1 backbone across the ABBA‐like motif, KEN, and D‐box binding site. The 2X‐CDCA3 mutant introduces a comparatively higher flexibility to the Cdh1 backbone in the linker region toward the D‐box and the ABBA‐like motif binding sites. Amongst the point mutants, pS222 + S229A and S222A + pS229, phosphorylated Ser229 increases the confirmational flexibility of the Cdh1 protein backbone compared to the phosphorylated Ser222. These findings highlight the role of phosphorylated residues on the structural stability of the protein backbone and suggest alternate binding of 2X‐CDCA3 mutant across the linker region.

### 2.3 Unbiased explicit solvent molecular dynamics of 2X and 4X mutants reveals alternate binding sites of ABBA motif mutants

**FIGURE 5:**
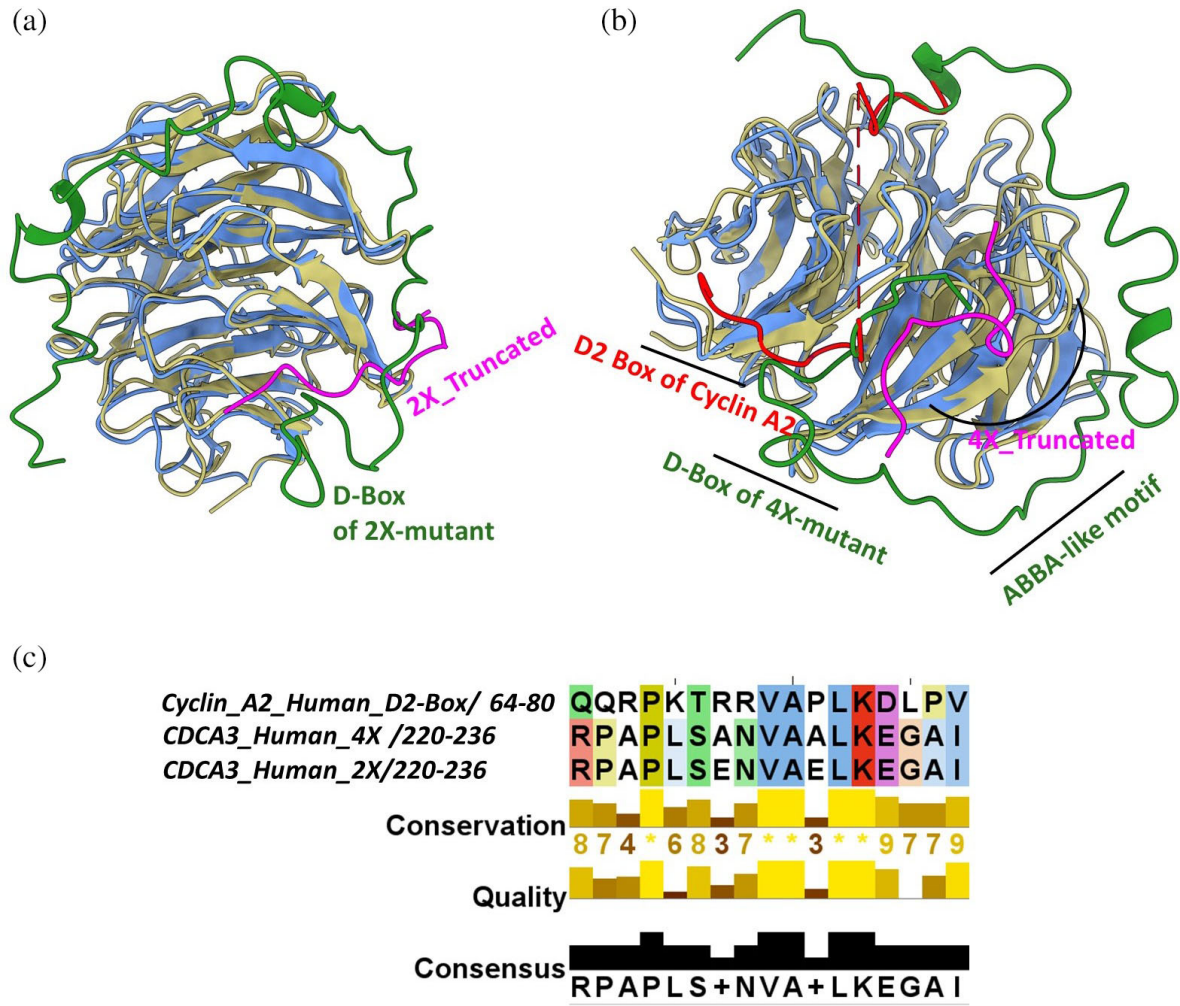
Interaction of C‐terminal ABBA domain peptide of CDCA3 2X and 4X mutants with Cdh1 after extended unbiased simulation. (a) Overlaid structures of the CDCA3‐Cdh1 complex with the full‐length C‐terminal domain of the CDCA3 2X mutant (green) simulated for 300 ns, and the 2X‐Truncated ABBA motif (Arg220‐Gly234) (pink) from CDCA3 simulated for 2 μs. The unbiased truncated CDCA3 peptide simulation demonstrates binding to the D‐box binding site of the Cdh1 WD40 domain (blue), (b) overlaid structures of the 4X‐Truncated peptide mutant (pink) and C‐terminal CDCA3‐4X mutant (green) simulated for 300 ns interacting with Cdh1 (blue) aligned with the crystal structure of human CyclinA2 (red) interacting with Cdc20 (brown). The 4X‐Truncated peptide after the extended simulation occupies a position near the D‐Box binding site of full‐length CDCA3 C‐terminal domain containing D‐box motif. Interestingly, this binding site is near the D2‐ box binding surface of Cyclin A2, (c) sequence alignment of the human CDCA3 ABBA‐like motif (220–236) of 4X and 2X mutants with the D2‐box motif of Cyclin A2 (64–80) shows conservation of the consensus D2‐box, explaining the binding of 4X‐Truncated and 2X‐Truncated peptide near the D box binding surface.

To investigate the difference between the MD simulation studies and the immunoprecipitation experiments, particularly that of 2X and 4X mutants, we performed long 2 μs unbiased MD simulations. We carried the simulation of the truncated ABBA‐like motif mutants (2X‐Truncated and 4X‐Truncated) in explicit solvent to identify their binding hotspots in Cdh1 WD40 domain. Clustering analysis using the DB SCAN algorithm (Shao et al. 2007) of both 2X and 4X truncated peptides in complex with Cdh1 from the simulation is displayed in Table 3. As shown in Figure 5a, the MD analyses of highest populated cluster identify 2X‐Truncated peptide bind to Cdh1, with the ABBA‐like motif occupying a site adjacent to the D‐box binding site. Similarly, the 4X mutant peptide (4X‐Truncated) simulation demonstrates that this peptide binds Cdh1 close to the D‐box binding site (Figure 5b). Detailed investigation of the 2X and 4X mutant sequences revealed that by mutating Ser229 and Glu230 to alanine, we created another D‐box‐like motif, similar to the D2‐box of human Cyclin A2 with consensus ^72^V‐X‐X‐L‐X‐D‐L‐X‐X‐N^81^ sequence (Figure 5c). We next compared the binding of Cyclin A2 and CDCA3 mutant peptides (2X and 4X) with their respective partners, Cdc20 and Cdh1. As shown in Figure 5b, our alignment of Cyclin A2 protein (bound to human Cdc20 [PDB ID: 6Q6H]) with the top cluster from the simulated trajectories of Cdh1‐CDCA3 4X‐Truncated peptide indicated a similar binding site for the CDCA3 ABBA‐like motif and the D2‐box of Cyclin A2 (Zhang et al. 2019b).

**TABLE 3:** Clustering analysis of the unbiased simulation of Truncated ABBA‐motif specific peptides of 2X and 4X mutants.

Simulation system	No. of frames in top cluster (C#0)	Percentage of frames in top cluster (%)	Average distance ± Stdev (Å)	Representative frame (#)	AvgCDist (Å)	DBI	pSF
2X‐Truncated	153,319	76.7	1.32 ± 0.51	120,252	3.62	1.46	14,599.79
4X‐Truncated	144,743	73	1.93 ± 0.43	89,504	5.65	0.46	175,261.67

To verify the above findings, we performed structure predictions of Cdh1 with 2X‐Truncated and Cdh1‐4X‐Truncated peptide sequences using AlphaFold2 multimer (Mirdita et al. 2022). While AlphaFold2 cannot predict phosphorylation dependent binding of proteins and peptides to partner biomacromolecules, it can reasonably recognize a hydrophobic patch in the cyclin‐A2 (AALAVL) and place it close to the true binding pocket on the cdca20 surface (AlphaFold2 2021). A similar approach was used in this manuscript to predict the binding of mutant peptides 2X and 4X on the Cdh1 surface. From Figure S6, it can be seen that the two peptides, 2X and 4X, bind to the D2‐box binding site of the CyclinA2. This observation further confirms the binding of 2X and 4X‐Truncated peptides at alternate binding site than the usual ABBA‐like motif‐binding site.

## 3 DISCUSSION

The APC/C coactivator proteins, Cdh1 and Cdc20, recognize their substrates via three specific disordered peptide regions: D‐box, ABBA‐motif, and KEN‐box, which bind to the WD40 repeat domain (He et al. 2013). Although human CDCA3 lacks a consensus ABBA motif, our data suggest that the ABBA‐like motif created by endogenous phosphorylation of S222 and S229 in the C‐terminus of CDCA3 by CK plays a crucial role to generate a short, linear motif (SLiM) degron and mediate an interaction with the APC/C modulator protein, Cdh1. Of the two‐phosphorylation sites, Ser222 phosphorylation mediates binding to Cdh1 via ionic interaction, while the phosphorylation of Ser229 induces structural changes towards the C‐terminus of ABBA‐like motif. Our findings highlight the importance of both helicity and ionic hydrophobic interactions for efficient binding between CDCA3 and Cdh1, as simultaneous mutation of Ser222 and Ser229 in CDCA3 abolishes its interaction with Cdh1.

IDPs are known to adopt different binding modes in presence of partner proteins or phosphorylation (i) disorder‐to‐order transitions leading to a well‐defined bound state, (ii) disordered binding leading to disordered bound states and (iii) fuzzy binding (Espinoza‐Fonseca et al. 2007; Fuxreiter 2020; Nicolaou et al. 2022). Herein, the results of MD simulations of various systems suggest that the CDCA3 C‐terminal domain bound to Cdh1 is a fuzzy complex wherein the KEN and D‐box motifs, at the C‐ and N‐termini of the sequence, respectively are clamped to the partner protein Cdh1. The di‐phosphorylated CDCA3 adopts a helical structure within the linker region (at the C‐terminus of ABBA‐like motif) when bound to Cdh1, which is lost upon double mutation in 2X CDCA3 mutant (Figure S1f), thus gaining higher flexibility at the disordered region and adapt different binding site (Figure 3a). The anchoring residues, widely studied for the molecular recognition in ordered proteins, also play a crucial role in stabilizing the transient structure and controlling the interactions between IDPs and their target protein partners (Huang and Liu 2011). We propose that upon di‐phosphorylation, CDCA3 binds to Cdh1 through an electrostatically driven induced fit mechanism and Ser229 stabilizes the structure, together acting as the anchor residues along with the KEN and D‐box motifs.

In addition to ionic interactions, it is worth noting that the hydrophobicity of the ABBA‐like motif also plays a crucial role in the interaction between the two proteins. Although, mutating Ser222 and Ser229 simultaneously to alanine disrupts the interaction between the Cdh1‐CDCA3, the incorporation of another alanine mutation (3X) creates hydrophobicity across the domains and change the protein flexibility, thus creating similar interaction profiles as the di‐phosphorylated system. The classical MD simulation data of 2X and 4X mutants did not align with the experimental findings due to the predominance of hydrophobic interactions in the ABBA‐like binding site, morphing of binding sites at the disordered region of CDCA3 due to absence of anchoring residues at the ABBA‐like motif. Considering that the 2X and 4X mutations enhance the structural flexibility of the linker region between D‐box and KEN domain, it is also possible that the binding entropy also contributes significantly to the binding free energy of CDCA3‐Cdh1. However, due to computational cost associated with the entropy calculation of highly flexible region, we have not considered the entropy factor towards the calculation of total binding energy. The unbiased MD simulations of truncated ABBA specific 2X‐Truncated and 4X‐Truncated peptides and the multimeric structural predictions generated with AlphaFold2 suggest their interactions with the alternate binding site of the Cdh1.

The sequence alignments of ABBA motif containing proteins show conservation throughout the species, for example, human CyclinA1, CyclinA2, BUB1, BUBR1, BUB1B, and Acm1 of budding yeast (He et al. 2013; Di Fiore et al. 2015; Kildey et al. 2021). However, these ABBA motifs bind preferentially to human Cdc20 or Cdh1 due to the residues at the ABBA binding site (the flanking region between the 2nd and 3rd blade) of the WD40 domain of Cdc20 or Cdh1 proteins (He et al. 2013; Di Fiore et al. 2015). Moreover, in human Cyclin A2, the presence of a second D‐box (referred to as D2‐box) from residue 72 to 81, except Val72 instead of Arg at position 1 in the consensus sequence of the canonical D‐box, creates a second binding mode. With the involvement of the KEN and D2‐box, this binding mode shows higher activity than the traditional binding mode with D1‐box, ABBA‐motif, and KEN domain. Surprisingly, in the Cyclin A2 of other species like rats and mice, the D1‐box is absent, and instead, a conserved D2‐box is present (Zhang et al. 2019b). This observation might also explain our immunoprecipitation analysis which identified a stronger association between Cdh1 and the 4X CDCA3 mutant where mutation has formed a D2‐like box. It is also worth noting that our *in vitro* immunoprecipitation analysis was performed using full‐length CDCA3. In contrast, the modeling and simulation studies were performed with the C‐terminal domain of CDCA3 bound to Cdh1 due to the computational limitations associated. As CDCA3 is intrinsically disordered, it is possible that N‐terminal regions of CDCA3 could impact its conformational flexibility and overall interaction with the coactivator protein, Cdh1. As such, the possible impact of other regions within CDCA3 and alternate binding positions might explain the discrepancy between *in silico* and *in vitro* observations for the 2X and 4X‐CDCA3 mutants.

## 4 CONCLUSION

In addition to the KEN and D‐box recognition motifs, our current study confirms the important contribution of phosphorylated anchoring residues within the ABBA‐like motif of CDCA3, to regulate the structural stability and interaction of this IDP with Cdh1. This study also highlights the contribution of ionic and hydrophobic residues that are required to promote Cdh1 binding and might be of importance, particularly for those other APC/C substrates which lack a classical ABBA motif.

## 5 MATERIALS AND METHODS

### 5.1 Immunoprecipitation assay of CDCA3 wild type and mutants

#### 5.1.1 Antibodies, reagents, and mammalian expression constructs

The CDCA3 antibody (HPA026587) and monoclonal FLAG M2 antibody (F1904) were purchased from Sigma Aldrich. Antibody against HA tag (#3724) was purchased from Cell Signaling Technology (Genesearch, Australia) and a complete EDTA‐free protease inhibitor mixture was purchased from Roche Applied Sciences. Donkey anti‐rabbit and anti‐mouse Alexa Fluor 488 antibodies were purchased from Life Technologies. The mammalian expression construct for HA‐Cdh1 was gifted by Marc Kirschner, and the CDCA3‐FLAG expression construct was generated by sub‐cloning full‐length CDCA3 sequence in pcDNA3.1+ using the BamHI and EcoRI restriction enzyme sites.

#### 5.1.2 Cell culture and cell treatments

The NSCLC cell lines (H460) were obtained from American Type Culture Collection (ATCC) and maintained in RPMI‐1640 medium +L‐glutamine (Life Technologies), supplemented with 10% foetal bovine serum (FBS, Sigma–Aldrich). The cells were cultured at 37°C in humidified 5% CO_2_ atmosphere.

#### 5.1.3 Collection of cell lysates, immunoprecipitation, and western blot analyses

For the collection of whole cell lysate, cells were washed with phosphate‐buffered saline (PBS) and lysed in lysis buffer (50 mM HEPES [pH 7.5], 150 mM KCl, 5 mM EDTA, 0.05% IGEPAL CA‐630 [v/v]), 1x protease inhibitor cocktail and 1x phosphatase inhibitor cocktail. Following sonication and centrifugation, total protein concentration was determined by Bicinchoninic Acid (BCA) Protein assay (Sigma–Aldrich). Total protein (20 μg) samples were denatured in 1x Laemmli Buffer supplemented with 8% β‐mercaptoethanol for 5 min at 80°C.

For immunoprecipitation assay, protein samples were prepared with 400 μg protein in 400 μL of lysis buffer. Lysates were incubated with 3 μg of HA antibody overnight at 4°C. Following incubation, lysates were incubated with protein A or G Dynabeads pre‐equilibrated with lysis buffer (Invitrogen). The Dynabeads were denatured using 2x Laemmli sample buffer supplemented with 8% β‐mercaptoethanol for 5 min at 80°C.

Samples were separated on Bolt 4–12% Bis‐Tris Plus pre‐cast gels (Life Technologies) and transferred onto nitrocellulose membrane (GE Healthcare Life Sciences) using the semi‐dry transfer Novex system (Life Technologies). Membranes first blocked using Odyssey blocking buffer (Li‐Cor) were incubated with primary antibody overnight at 4°C in a 1:1 solution of Odyssey blocking buffer and PBS‐T. All primary antibodies were used at a dilution of 1:1000 except for antibodies targeting CDCA3 (1:800). Following incubation, membranes were washed with PBS‐T and incubated with appropriate secondary antibodies and visualized using the Li‐Cor Odyssey system. Images were acquired and subjected to densitometric analysis.

### 5.2 Molecular modeling and simulation of CDCA3‐Cdh1 Protein‐Peptide complex

#### 5.2.1 Construction of CDCA‐Cdh1 model

The molecular modeling of WT CDCA3‐Cdh1 was performed as per the method described in our previous publication (Kildey et al. 2021). The KEN, D‐Box, and non‐canonical ABBA motifs at the C‐terminus of human CDCA3 (Ser193‐Glu267) were built to interact with the WD40 propeller domain of human Cdh1 protein molecule (Ser172‐Ser474). The N‐terminal domain of CDCA3 was not considered for modeling due to its intrinsically disordered nature and non‐homology with known 3D protein structure. The model was built based on the 3D structure of APC/C activator protein, Cdh1 of *Saccharomyces cerevisiae* bound to the three degrons of APC/C‐Cdh1 modulator 1 (Acm1, PDB ID: 4BH6) (He et al. 2013). The KEN and D‐box motifs of APC/C‐Cdh1 modulator 1 were aligned with the homologous sequences at the C‐terminus of CDCA3. The motif consisting of Arg220‐Leu231 sequence mimicked the ABBA motif of Acm1 and was designated as non‐canonical ABBA‐like motif. Position restraints were used to model the degrons motifs followed by modeling remaining residues from the CDCA3 template using the Modeler tool of UCSF Chimera v1.2.5 (Pettersen et al. 2004). Due to the structural homology of *S. cerevisiae* Cdh1 with human Cdh1 (PDB ID: 4UI9, Chain R), it was finally replaced using the MatchMaker tool of UCSF Chimera (Pettersen et al. 2004; Chang et al. 2015). Other peptides (pS222 + S229A, S222A + pS229, S222A, S229A, 2X, 3X, and 4X) were modeled by mutating the specific amino acid residues of the WT sequence Chimera v1.5 (Pettersen et al. 2004).

#### 5.2.2 Molecular dynamics simulation of wild type and mutant CDCA3‐Cdh1 complex

MD simulations were performed on the modeled WT CDCA3‐Cdh1 complex and CDCA3‐mutants with Cdh1. The tleap program of AmberTools 16 package (Case RMB et al. 2016) was used to prepare the input files for MD simulation. The serine residues at position 222 and 229 of CDCA3 were phosphorylated. The AMBER force fields ff99SB‐ILDN (Lindorff‐Larsen et al. 2010), Phosaa10 (Steinbrecher et al. 2012), and TIP3P (Abriata and Dal Peraro 2021) were used to parameterize the unphosphorylated proteins, phosphorylated proteins and water molecules, respectively. The protein–protein complex was placed into a truncated octahedral solvent box with a nearest distance of less than 12 Å between the protein and the box boundary and solvated using explicit water. Either sodium or chloride ions were used to neutralize the system, followed by further addition of equimolar quantities of Na^+^ and Cl^−^ ions to get the final salt concentration of 0.25 M. All the covalent bonds with hydrogen were constrained using the SHAKE algorithm (Ryckaert et al. 1977), and Langevin dynamics (Bussi and Parrinello 2007; Quigley and Probert 2004) were applied to control the temperature. The long‐range electrostatic interactions were evaluated using Particle Mesh Ewald (PME) method (Darden et al. 1993). All MD simulations were carried out using the PMEMD.CUDA module (Salomon‐Ferrer et al. 2013; Gotz et al. 2012) of AMBER 16. The non‐bonded interaction cut‐off was set to 12 Å during energy minimization and simulation steps. Initial energy minimization of the system was performed for 1000 steps using the steepest descent and conjugate gradient method for another 500 steps. The optimized structure was then heated from 0 to 300 K in a 50 picosecond (ps) simulation by applying a 10 kcal/mol/Å^2^ positional harmonic restraint to protein and ligand atom coordinates using a Berendsen thermostat (Berendsen et al. 1984) with constant particle number, volume, and temperature (NVT) ensemble. Each system was subjected to further equilibration in a constant particle number, pressure, and temperature (NPT) ensemble maintained at 1 atm and 300 K for 650 ps with restraints as per the NVT run. Another 20 nanoseconds (ns) of equilibration was performed by applying a harmonic position restraint of 2 kcal/(mol Å^2^) on KEN, D‐box, and the non‐canonical ABBA motif under the same condition. A constant pressure period boundary condition was used with an average pressure of 1 atm with a non‐bonded cut‐off of 12 Å. The system's pressure was maintained using a Berendsen barostat with a relaxation time of 1 ps. The system temperature was maintained at 300 K using the Langevin thermostat with a collision frequency of 5.0 ps^−1^ (Quigley and Probert 2004). After every 5000 steps, the simulation restart files and energy information were written to mdout and mdinfo files, coordinates and the velocities were written to the trajectory files. Another 100 ns long equilibration was performed with NPT ensemble. A final 300 ns production run with a timestep of 1 femtoseconds (fs), was carried out using the NPT ensemble to study the stability and the conformational changes in the complex. The simulation of each system was repeated twice starting from a 100 ns equilibration followed by a 300 ns production run using the same parameters. System coordinates were written out at 10 ps intervals during the production runs. The analyses and relative free energy calculations were carried out on trajectories of the final 300 ns (30,000 frames) using the CPPTRAJ module (Roe and Cheatham III 2013) of AmberTools 16.

#### 5.2.3 Calculation of relative free energy of binding using MM‐GBSA and pairwise decomposition of residue interaction energies

The Molecular Mechanics‐Generalized Born Solvent Area (MM‐GBSA) relative binding free energy on MD trajectory containing the last 30,000 frames were calculated for each protein–protein complex using the MMPBSA.py module (Miller III et al. 2012) from the Amber16 package. Residue‐based pairwise free energy decomposition calculations were performed using the “idecomp = 1” option in the Sander module of the AMBER program. Moreover, the energy components from Van der Waals, electrostatic energy, polar, and SAS parts of the GB terms were produced using the “idecomp = 3” option in the AMBER package. The GB^OBC‐II^ (Onufriev et al. 2000; Onufriev et al. 2004) implicit solvent model (igb = 5, PBRradii = mbondi2) was used to calculate the polar solvation energy component, and the ionic strength of the systems were set to 25 mM. The default dielectric constant (ε) of 1.0 and 80.0 were used for solute and solvent, respectively. The non‐polar component of solvation was calculated using solvent accessible surface area (SASA) based on the LCPO algorithm (Weiser et al. 1999). Detailed calculations for binding free energetics are discussed in Appendix S1.

#### 5.2.4 Unbiased enhanced molecular dynamics simulation of 2X and 4X mutant of ABBA‐like motifs using explicit solvent

The unbiased MD simulation of protein–protein interactions in explicit water have been used to investigate their binding states and mechanism (Milić et al. 2018; Tajne et al. 2012). A similar approach has been used here to examine the binding of 2X and 4X mutant peptides of CDCA3 on the Cdh1 surface, summarized in Appendix S1.

#### 5.2.5 Analysis and visualization of molecular dynamics trajectories

Clustering analyses of all the simulation systems were performed to populate similar conformational ensembles from the MD simulation trajectories into smaller subsets. The density‐based spatial clustering of application with noise (DB SCAN) algorithm (Shao et al. 2007) was used to perform this analysis using the OpenMP (OMP) version of CPPTRAJ (Roe and Cheatham III 2018). The minpoints and the epsilon values for the clustering analysis using the DB‐Scan algorithm were defined by generating the K‐dist plot. For this analysis, the ions and the solvents were ignored. The conformational changes in the Cdh1 protein structures were accessed by measuring the RMSFs of backbone atoms of the Cdh1 structures from the simulation trajectories using CPPTRAJ. The change in secondary structure of the CDCA3 peptide across the D‐box, ABBA‐like motif, and KEN domain upon phosphorylation was evaluated by performing secondary structure prediction analysis using the Dictionary of Secondary Structure of Proteins (DSSP) algorithm (Kabsch and Sander 1983). UCSF ChimeraX v1.5 was used to visualize and prepare images of the representative conformations obtained from the top cluster of MD trajectories (Pettersen et al. 2021).

## AUTHOR CONTRIBUTIONS

**Tabassum Khair Barbhuiya:** Conceptualization (equal); data curation (lead); formal analysis (lead); investigation (equal); methodology (equal); software (lead); validation (lead); visualization (lead); writing – original draft (lead); writing – review and editing (equal). **Mark Fisher:** Formal analysis (supporting); visualization (supporting); writing – review and editing (equal). **Eric D. Boittier:** Methodology (supporting). **Emma Bolderson:** Writing – review and editing (supporting). **Kenneth J. O'Byrne:** Resources (equal); writing – review and editing (supporting). **Derek J. Richard:** Funding acquisition (equal); resources (equal); writing – review and editing (equal). **Neha S. Gandhi:** Conceptualization (equal); funding acquisition (equal); project administration (equal); resources (equal); supervision (equal); validation (equal); writing – review and editing (equal).

## CONFLICT OF INTEREST STATEMENT

The authors declare no conflicting financial interests; Emma Bolderson, Derek J. Richard, and Kenneth J. O'Byrne are founders of Carpe Vitae Pharmaceuticals. Emma Bolderson, Mark Nathaniel Adams, Kenneth J. O'Byrne, and Derek J. Richard are inventors on provisional patent applications filed by Queensland University of Technology.

## Supporting information


**Appendix S1:** Supporting Information

## Data Availability

The simulation data supporting the findings of this study are available in the supplementary material of this article.
