# Supplementary material for: Structural investigation of CDCA3‐Cdh1 protein–protein interactions using in vitro studies and molecular dynamics simulation
Source: Protein Sci. 2023 Feb 14;32(3):e4572. doi: 10.1002/pro.4572 (PMC9926468; doi:10.1002/pro.4572)
Supplement: Supplementary file 1 — Appendix S1: Supporting Information [file PRO-32-e4572-s001.docx]

**Supplementary Materials**

**for**

**Structural Investigation of CDCA3-Cdh1 Protein-Protein Interactions using *in-vitro* Studies and Molecular Dynamics Simulation**

**Supporting Figures**


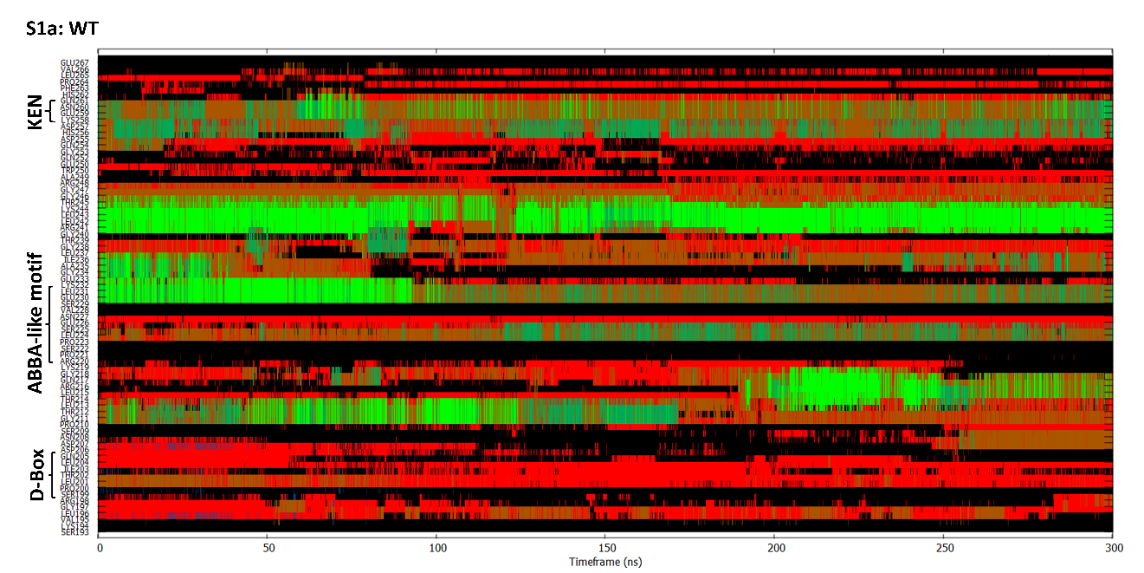


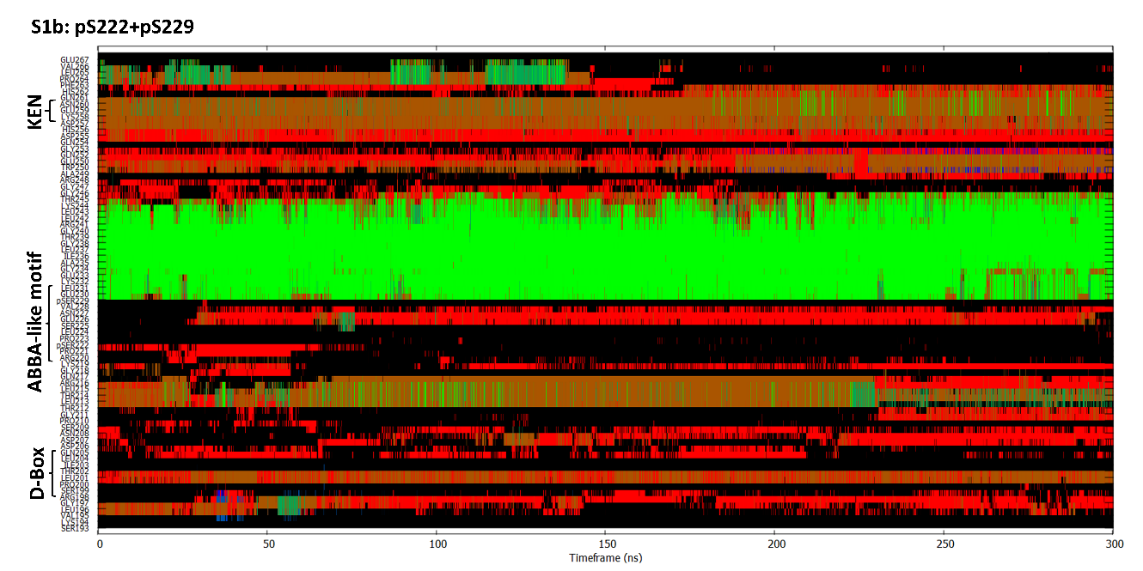


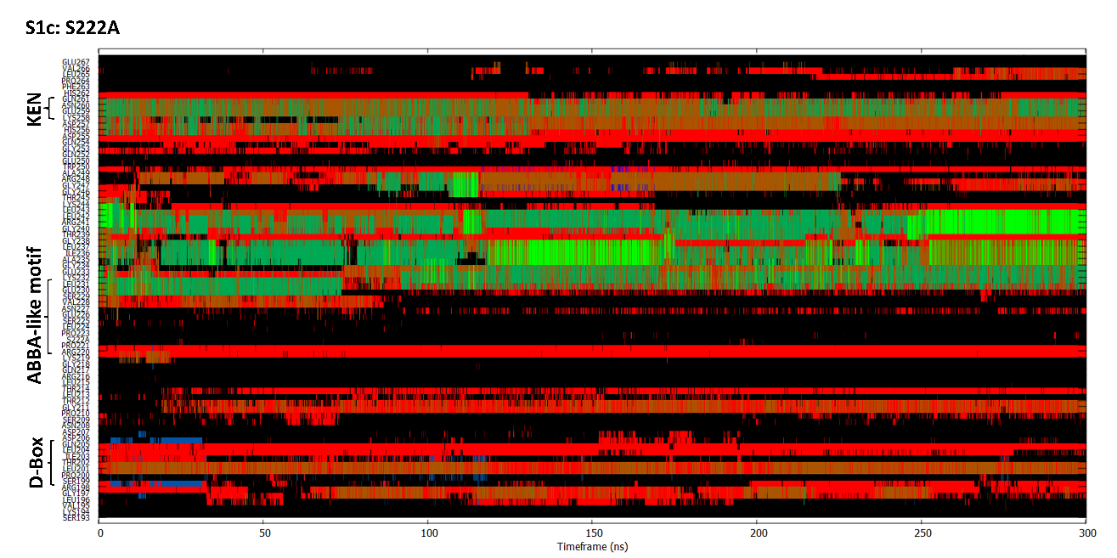


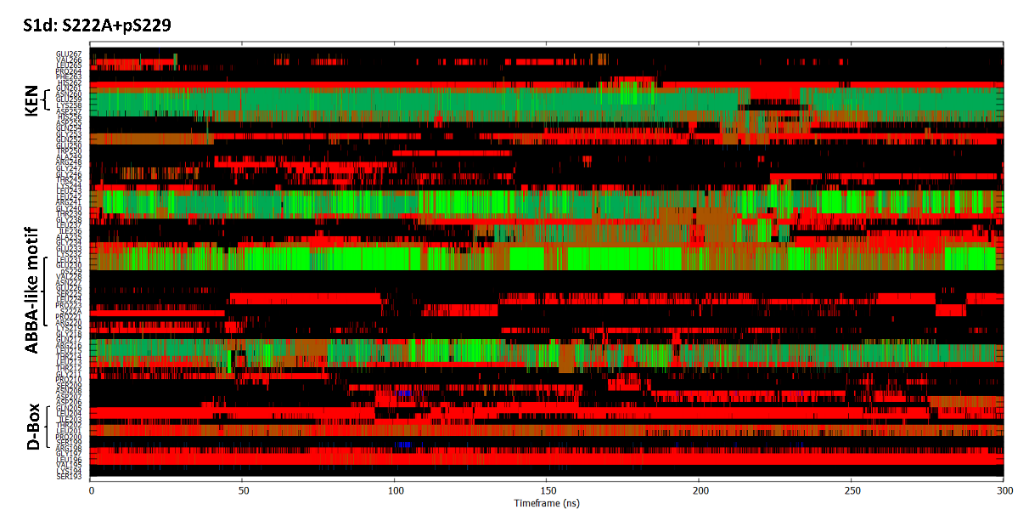


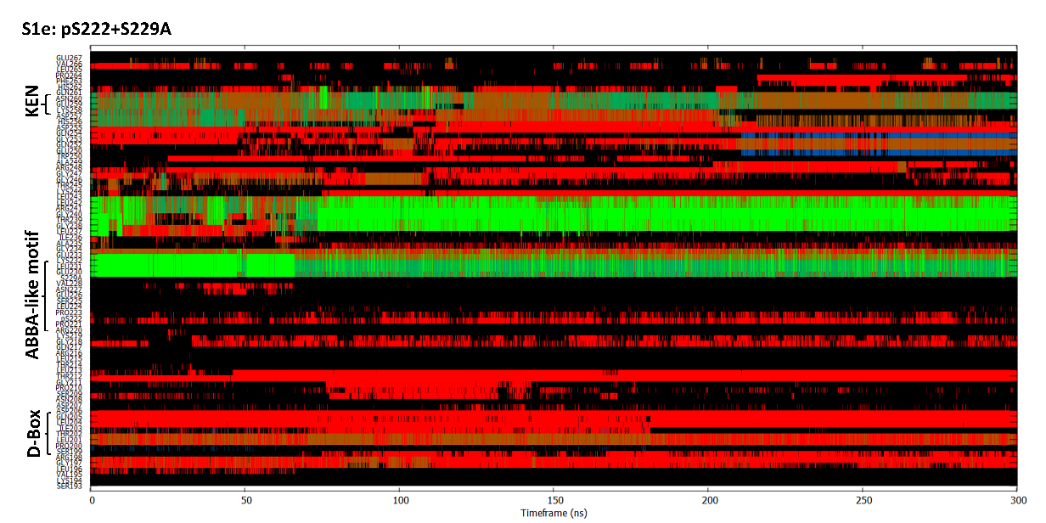


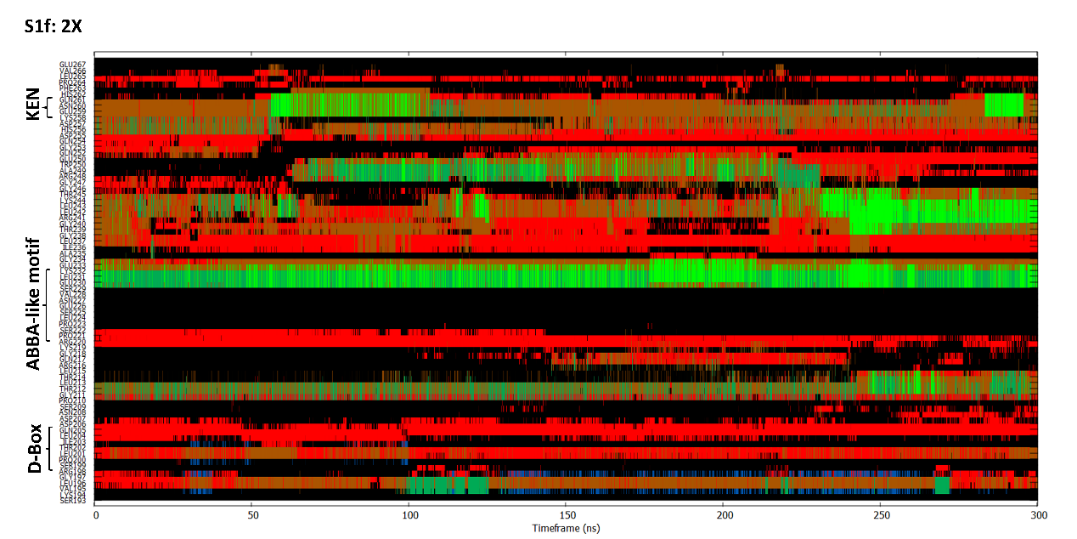

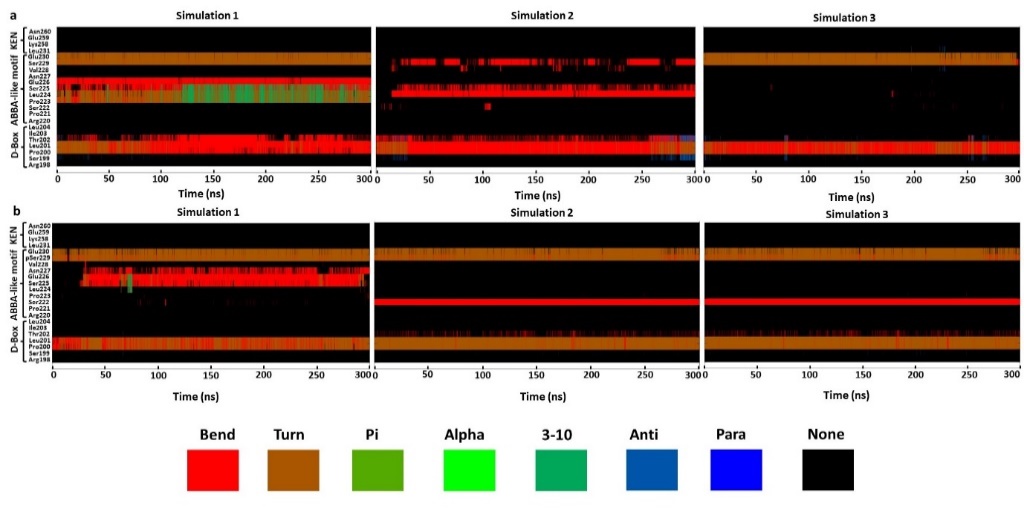


**Figure S1a-f: Secondary structure propensities of the WT-CDCA3, the CDCA3-phospho-mutants, and 2X CDCA3 N-terminal domain bound to Cdh1**

*The secondary structure estimation of CDCA3 C-terminal peptides along the D box (Arg198-Leu204), ABBA-like motif (Agr220-Leu231) and KEN domain (Lys258-Asn260) from the MD simulation trajectories of 30,000 frames using DSSP algorithm implemented using cpptraj in AMBER.* ***1a)*** *WT-CDCA3 shows an unstructured form with some structural bends and turns throughout the C-terminal domain and a small transient alpha-helical structure,* ***1b)*** *The di-phosphorylated CDCA3, pS222+pS229, shows the formation of prominent alpha-helical structure starting at pSer229 residue in ABBA-like motif throughout the simulation,* ***1c)*** *the S222A simulation shows some structural bends, 1d) S222A+pS229 induces some alpha-helical propensities* ***1e)*** *pS222+S229A has small helical propensities across the domain,* ***1g)*** *2X CDCA3 mutant showed the secondary structure profile similar to the unphosphorylated WT-CDCA3.*


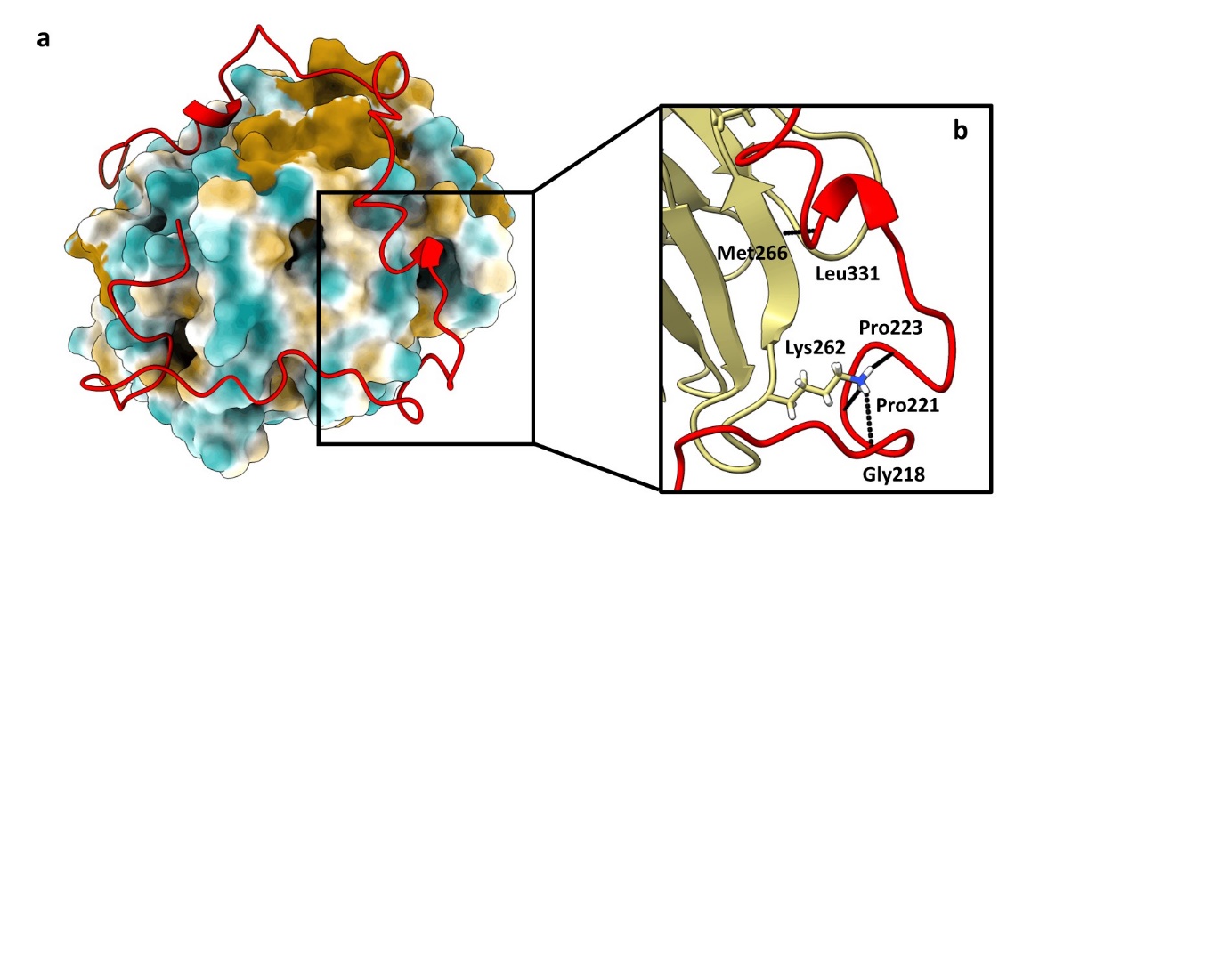


**Figure S2: Interaction of CDCA3 3X mutant with the Cdh1 Surface**

***a:*** *The interaction of the 3X mutant peptide of CDCA3 with the molecular lipophilic potential (mlp) surface (coloured from dark cyan: most hydrophilic to white to dark goldenrod: most lipophilic) of Cdh1 WD40 domain shows a predominance of hydrophobic interactions.* ***b:*** *The protein-peptide interacting residues form H-bond interactions with Lys262 at the ABBA-binding interface. Images are prepared from the top cluster of the simulation trajectories using ChimeraX v1.5*


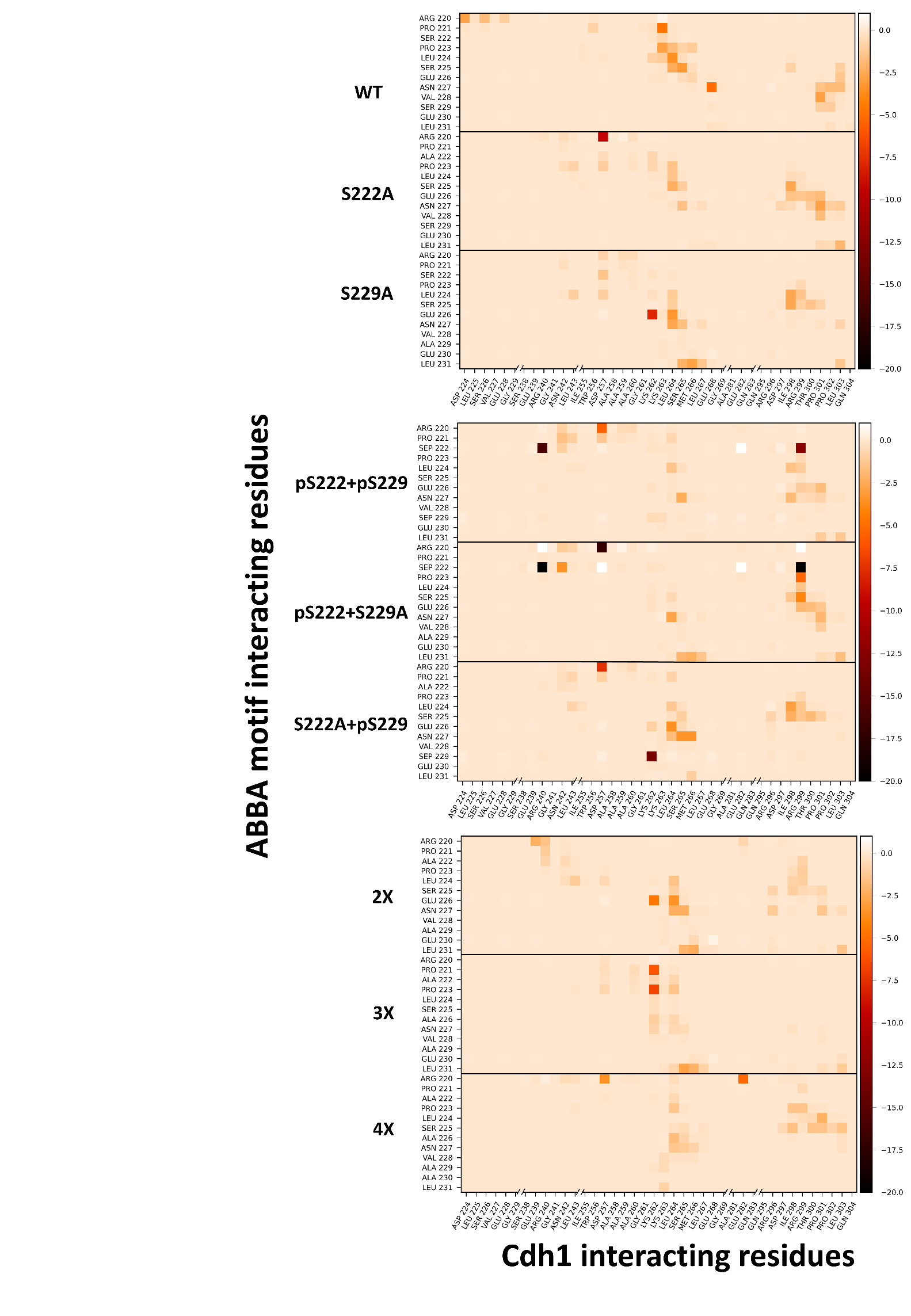


***Figure S3:*** ***Pairwise energy decomposition of CDCA3 C-terminus ABBA-like motif residues (WT and mutants) interacting with Cdh1 WD domain***

*Heatmap of pairwise energy decomposition from MM-GBSA binding energy calculations of Cdh1 interacting domains with the residues of CDCA3 and its mutants along the ABBA-like motifs. The residues of Cdh1 not participating in interaction or below energy cut-off of -1.4 kcal/mol are not considered in the heatmap, hence indicated as “//” on the X-axis. The 3^rd^ dimension of heatmap represents energies in kcal/mol.*

*
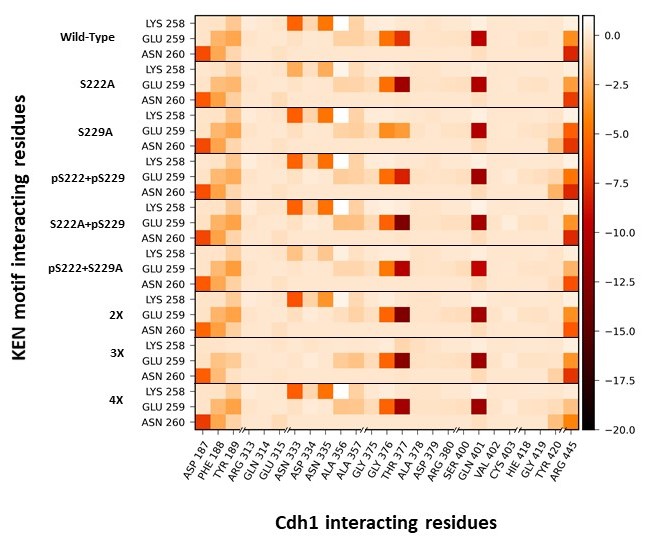
*

***Figure S4:*** ***Pair-wise energy decomposition of KEN domain residues of CDCA3 (WT and mutants) interacting with Cdh1 WD domain***

*Heatmap of pairwise energy decomposition from MM-GBSA binding energy calculations of Cdh1 interacting domains with the KEN domain of WT-CDCA3 and its alanine mutants. The residues of Cdh1 not participating in interaction or below energy cut-off of -1.4 kcal/mol are not considered in the heatmap, hence indicated as “//” on the X-axis. The 3^rd^ dimension of the heatmap represents energies in kcal/mol.*

*
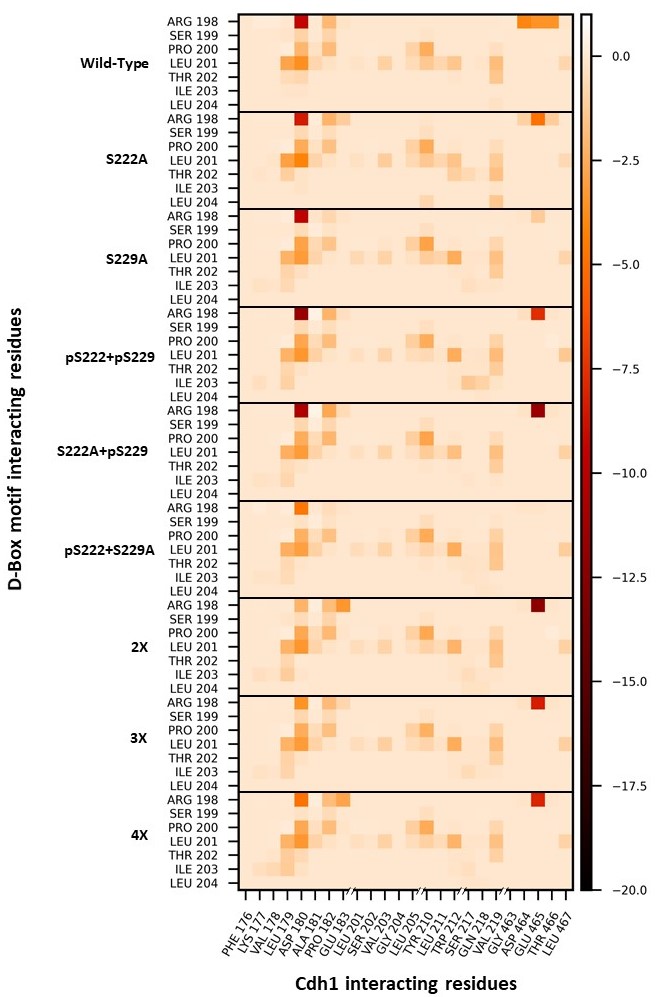
*

***Figure S5:*** ***Pairwise energy decomposition of D-box motif residues of CDCA3 (WT and mutants) interacting with Cdh1 WD domain***

*Heatmap of pairwise energy decomposition from MM-GBSA binding energy calculations of Cdh1 interacting domains with the D-box motif of WT-CDCA3 and its alanine mutants. The residues of Cdh1 not participating in interaction or below energy cut-off of -1.4 kcal/mol are not considered in the heatmap, hence indicated as “//” on the X-axis. The 3^rd^ dimension of heatmap represents energies in kcal/mol.*

*
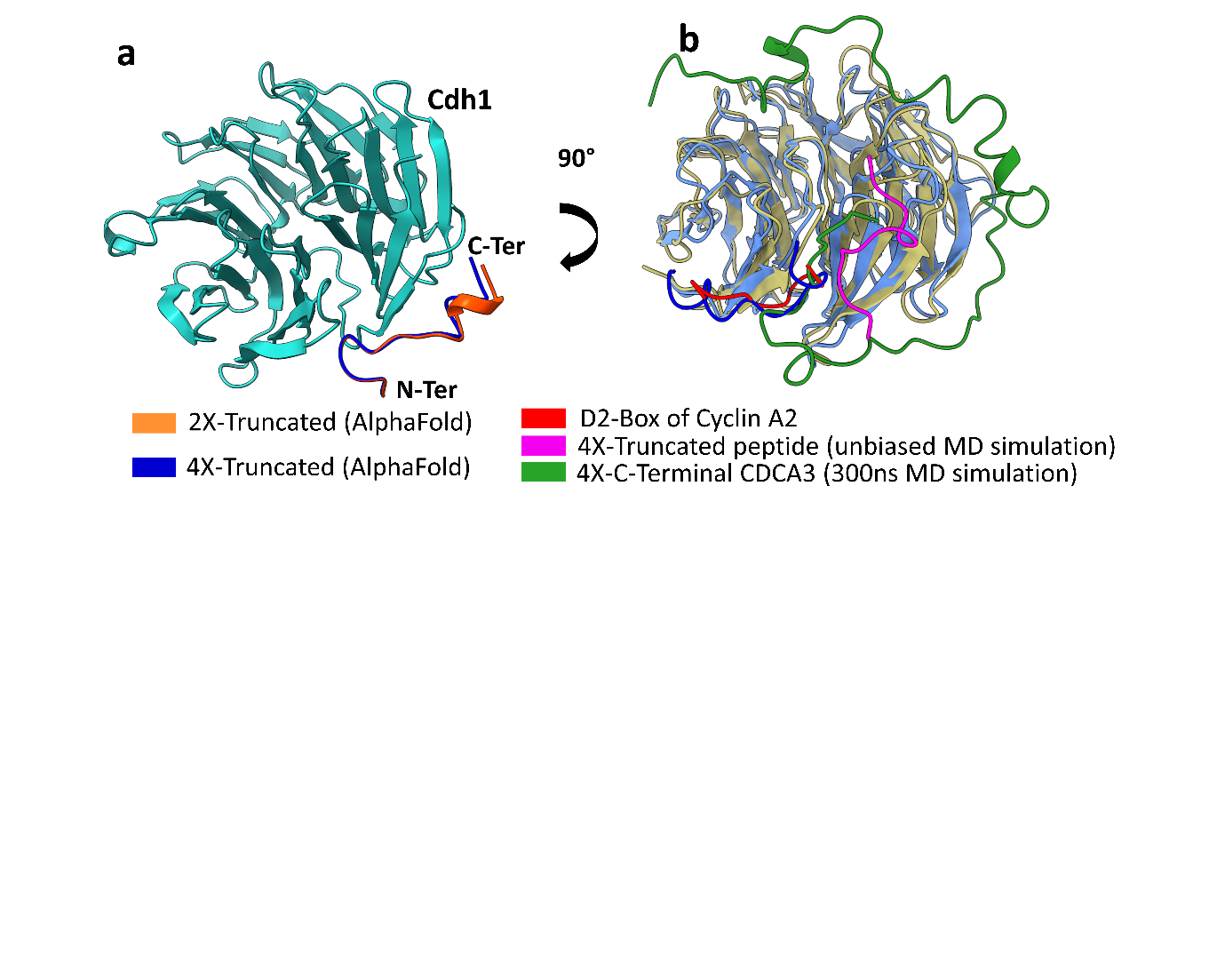
*

***Figure S6:*** ***AlphaFold2 multimeric structure prediction of 2X-Truncated and 4X-Truncated peptide complexed with Cdh1***

***a:*** *AlphaFold2 prediction of 2X-Truncated and 4X-Truncated peptides showed similar binding sites on the Cdh1 binding surface,* ***b:*** *The overlaid diagram of Cdh1-4X-Truncated peptide from AlphaFold2 prediction aligned with the CDCA3-4X domain from 300 ns simulation and Cdc20 bound Cyclin A2. The 4X-Truncated peptide from AlphaFold prediction binds at the D2-Box binding site of CyclinA2, which is near the binding site of the 4X-Truncated peptide from the most populated cluster of 2µs extended simulation and D-box of CDCA3-4X C-terminal domain from 300 ns simulation.*

*
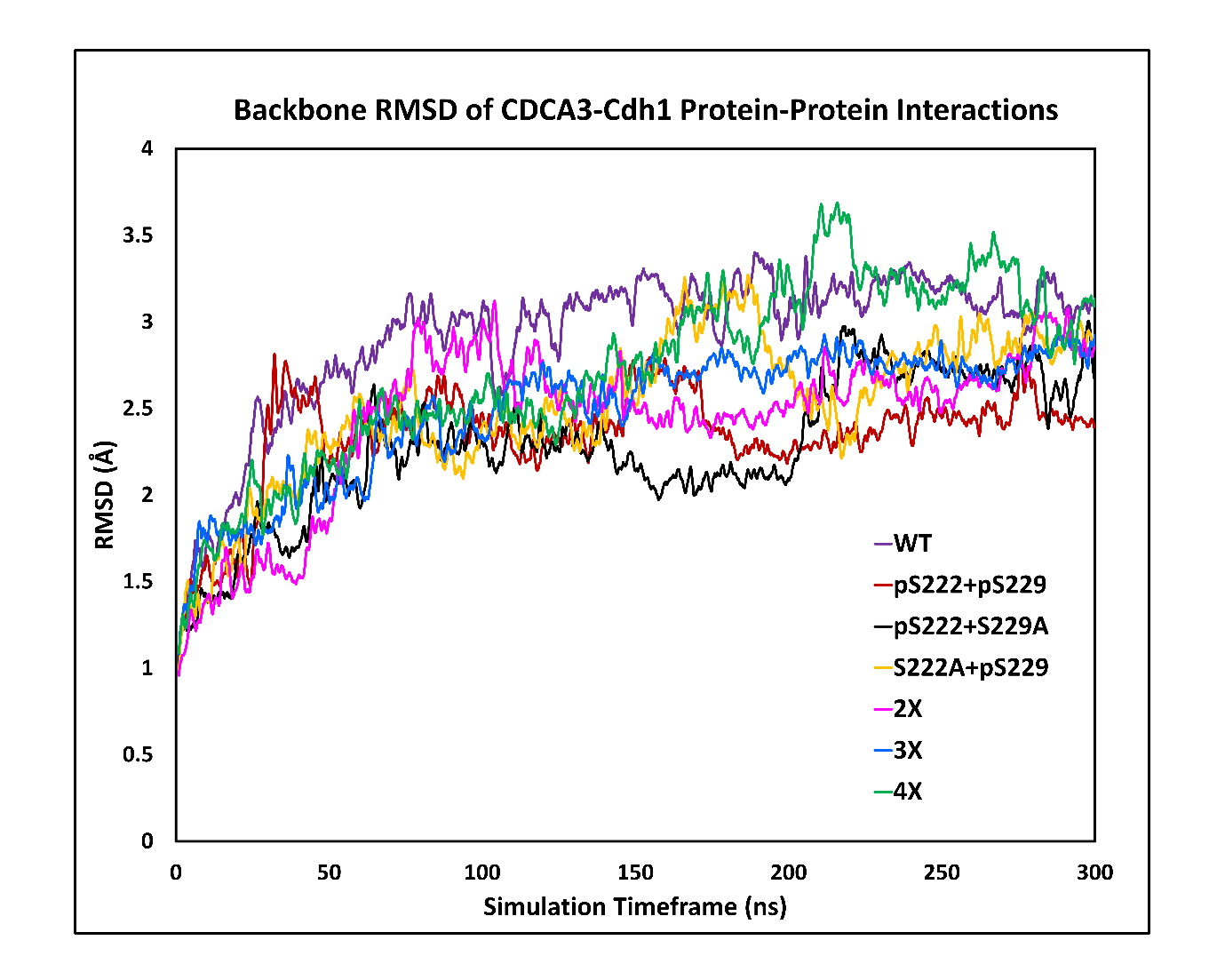
*

***Figure S7:*** ***Root mean-square deviation (RMSD) time plot (moving average) of the C-terminal CDCA3-Cdh1 protein-protein interactions***

**Supporting Materials and Methods**

**Free Energy calculation using MM-GBSA**

The binding free energies (ΔG_binding_) calculation for MM-GBSA in kcal/mol considers the basic thermodynamic Gibb’s Free energy equation (1):s

$\boldsymbol{\Delta G= \Delta H-T\Delta S}$ ***(1)***

Where, ΔG is the enthalpy change while T and ΔS are the temperature and change in entropy of the system, respectively. Therefore, the binding free energy for each protein-peptide complex can be calculated by determining the change in free energies of the bound complex and the unbound form of each component, given by the following equation (2):

***ΔG_binding_ = G_CDCA3-Cdh1_ – (G_CDCA3_ + G_Cdh1_) (2)***

The free energies of each component, i.e., the peptide, protein, and complex constitute the contribution from the following three different energy components mentioned in equation (3):

***G = (E_MM_ + E_Sol_) – TΔS (3)***

where, the first two terms i.e., *E_MM_* and *E_Sol_* are the enthalpy terms. *E_MM_* is the molecular mechanics energy in a gaseous state comprising the contribution from energies of bonded (bond stretch, bond angle, dihedral angle, torsions) and non-bonded interactions components (van der Waals, electrostatic and internal energies) as per equation (4):

***E_MM_ = E_bonded_ + E_non-bonded_ (4)***

***E_sol_ = E_GB/PB_ + E_SASA_  (5)***

In equation (5), *E_sol_* represents the solvation energies arising from the contribution of the implicit polar electrostatic solvation component (E_GB/PB_) as well as the non-polar contribution (E_SASA_) from non-electrostatic solvation components, estimated by GB/PB models and SASA, respectively. TΔS is the contribution from temperature and change in entropy upon binding. We have not considered the entropy term because of the high computational cost and low prediction accuracy associated with the macromolecular system.

### Unbiased enhanced MD simulation of 2X and 4X mutant of ABBA-like Motifs using explicit solvent

In the modelled Cdh1 structure, the CDCA3 ABBA-like motif containing sequence (Arg220-Gly334) was placed randomly at a 10Å distance from the Gln185-Leu225 of the Cdh1. Alanine mutants were made at their respective positions for 2X and 4X mutants using UCSF Chimera v1.14. These coordinates were used to prepare the input files for unbiased MD simulation of these two systems using the tleap program of AmberTools 16. The AMBER force field, ff14SB for protein (1) and TIP3P water force fields were used. The protein-peptide complex was solvated with TIP3P water molecules in a truncated octahedral solvent box of 14Å. 10 Na^+^ and Cl^-^ ions were added to each system, and additional counterions were added to the simulation system to neutralise the overall net charge of the system. The long-range electrostatic interactions were evaluated using the Particle Mesh Ewald (PME) method. The non-bonded interaction cut-off was set to 12 Å during energy minimisation and simulation steps. An initial 500 steps of steepest descent minimisation were performed on the simulation system by applying positional restraints on the proteins with a force constant of 100 kcal/mol/Å^2^. The whole system was then minimised with 500 cycles of steepest descent and 500 cycles of the conjugate gradient method. The minimised system was heated from 0 to 300 K with an NVT ensemble and positional restraints on the protein using a force constant of 10.0 kcal/mol/Å^2^. The system was relaxed at 300K in the NPT ensemble for 0.1 ns restraining the protein-heavy atoms using a weaker force constant of 2.0 kcal/mol/Å^2^. Again, the system was relaxed at 300K for 0.5 ns in the NPT ensemble, followed by a 20 ns equilibration. Finally, a 2 microsecond (µs) production simulation with a stepsize of 2 fs was carried out in an NPT ensemble. The periodic boundary condition was maintained at a constant pressure of 1 atm, and the non-bonded interaction cut-off was set to 12 Å. The pressure of the system was maintained using the Berendsen barostat with a relaxation time of 1 ps. The system temperature was maintained at 300K using the Langevin thermostat with a collision frequency of 5.0 ps^-1^ (2). After every 5000 steps, the simulation restart files and energy information were written in mdout and mdinfo files, coordinates and the velocities were written to the trajectory files. The trajectories of the complete 2 µs simulation, consisted of 200,000 frames captured at an interval of 10 ps using the CPPTRAJ module (3) of AmberTools 16 for further analyses.

### Electrostatic potential surface

The electrostatic potential surface for the Cdh1 WD domain was calculated using the Adaptive Poisson-Boltzmann Solver (APBS) (4). The Cdh1 structure was prepared using automatic PDB2PQR software followed by APBS calculations. Default parameters were used for APBS calculation, except that the temperature of the system and the ionic strength were set to 300 K, and 0.25M, respectively.

### Sequence Alignment and AlphaFold2 Prediction

The sequence alignment of the ABBA-like motif of 2X and 4X mutant (Arg220-Ile236) with the D2-box of Cyclin A2 (Gln64-Val80) was performed using the Clustal Omega multiple alignment webserver (5) and visualised using Jalview v2.11.2.3 multiple sequence alignment editor and analysis platform (6). The structures of the 2X-Truncated and 4X-Truncated peptides complexed with Cdh1 were predicted by AlphaFold-Multimer using the open-source, ColabFold, an optimised version of AlphaFold2 (7) in ChimeraX v1.5 (8, 9).

### References

1. Maier JA, Martinez C, Kasavajhala K, Wickstrom L, Hauser KE, Simmerling C (2015) ff14SB: improving the accuracy of protein side chain and backbone parameters from ff99SB. Journal of chemical theory and computation 11:3696-3713.

2. Quigley D, Probert M (2004) Langevin dynamics in constant pressure extended systems. The Journal of chemical physics 120:11432-11441.

3. Roe DR, Cheatham III TE (2013) PTRAJ and CPPTRAJ: software for processing and analysis of molecular dynamics trajectory data. Journal of chemical theory and computation 9:3084-3095.

4. Jurrus E, Engel D, Star K, Monson K, Brandi J, Felberg LE, Brookes DH, Wilson L, Chen J, Liles K (2018) Improvements to the APBS biomolecular solvation software suite. Protein Science 27:112-128.

5. Sievers F, Higgins DG. The clustal omega multiple alignment package. (2021) Multiple sequence alignment. Springer, pp. 3-16.

6. Waterhouse AM, Procter JB, Martin DM, Clamp M, Barton GJ (2009) Jalview Version 2—a multiple sequence alignment editor and analysis workbench. Bioinformatics 25:1189-1191.

7. Mirdita M, Schütze K, Moriwaki Y, Heo L, Ovchinnikov S, Steinegger M (2022) ColabFold: making protein folding accessible to all. Nature Methods:1-4.

8. Pettersen EF, Goddard TD, Huang CC, Meng EC, Couch GS, Croll TI, Morris JH, Ferrin TE (2021) UCSF ChimeraX: Structure visualization for researchers, educators, and developers. Protein Science 30:70-82.

9. Goddard TD, Huang CC, Meng EC, Pettersen EF, Couch GS, Morris JH, Ferrin TE (2018) UCSF ChimeraX: Meeting modern challenges in visualization and analysis. Protein Science 27:14-25.
